# Interactions between microbiota and cervical epithelial, immune, and mucus barrier

**DOI:** 10.3389/fcimb.2023.1124591

**Published:** 2023-02-24

**Authors:** Mengting Dong, Yalan Dong, Junyi Bai, Huanrong Li, Xiaotong Ma, Bijun Li, Chen Wang, Huiyang Li, Wenhui Qi, Yingmei Wang, Aiping Fan, Cha Han, Fengxia Xue

**Affiliations:** ^1^ Department of Obstetrics and Gynaecology, Tianjin Medical University General Hospital, Tianjin, China; ^2^ Tianjin Key Laboratory of Female Reproductive Health and Eugenic, Department of Gynecology and Obstetrics, Tianjin Medical University General Hospital, Tianjin, China

**Keywords:** cervicovaginal microbiota, uterine cervix, epithelial, immune, mucus, chlamydia trachomatis, human papilloma virus, human immunodeficiency virus

## Abstract

The female reproductive tract harbours hundreds of bacterial species and produces numerous metabolites. The uterine cervix is located between the upper and lower parts of the female genital tract. It allows sperm and birth passage and hinders the upward movement of microorganisms into a relatively sterile uterus. It is also the predicted site for sexually transmitted infection (STI), such as Chlamydia, human papilloma virus (HPV), and human immunodeficiency virus (HIV). The healthy cervicovaginal microbiota maintains cervical epithelial barrier integrity and modulates the mucosal immune system. Perturbations of the microbiota composition accompany changes in microbial metabolites that induce local inflammation, damage the cervical epithelial and immune barrier, and increase susceptibility to STI infection and relative disease progression. This review examined the intimate interactions between the cervicovaginal microbiota, relative metabolites, and the cervical epithelial-, immune-, and mucus barrier, and the potent effect of the host-microbiota interaction on specific STI infection. An improved understanding of cervicovaginal microbiota regulation on cervical microenvironment homeostasis might promote advances in diagnostic and therapeutic approaches for various STI diseases.

## Introduction

Cervicovaginal microbiota have a mutual relationship with the host. The lower genital tract of women provides an ecological niche for bacterial colonisation. *Lactobacillus* spp. predominates the microbial flora in a healthy female genital tract, which confers several benefits to the host. It ferments the glucose and maltose of the vaginal epithelial cells to produce lactic acid which maintains vaginal pH at 3.8~4.5. Lactic acid and its associated acidic microenvironment help regulate inflammation. *Lactobacilli* also secrete bacteriocin (Scillato et al., 2021), which inhibits the proliferation of other microorganisms and has strong epithelial cell adhesion ability, thereby competing with pathogens for living space and nutrient uptake (Kwok et al., 2006). Female genital microbiota can be tightly regulated by host factors such as age, menstrual cycle, sex steroid hormones, race, living habits, immunity, and genetic polymorphism. Microbial metabolites, components, and bacteria can interact with host epithelial cells and resident immune cells, leading to alterations in the local ecosystem and affecting defences against pathogen infection and disease progression. The dominant *Lactobacilli* flora maintains the health of the reproductive tract, whereas dysbiosis of the microbiota, wherein Lactobacilli are significantly reduced or absent and replaced by pathogenic bacteria is indicative of the development of reproductive tract-associated diseases (Smith and Ravel, 2017).

The female reproductive tract (FRT) can be divided into the lower FRT (vagina and ectocervix) which contains a large microbial presence, and the upper FRT (endocervix, uterus, and oviduct) which is relatively sterile. The cervix includes the ecto- and endocervix and plays a specific role in FRT. On the one hand, the cervix acts as a channel through which human sperm can migrate into the uterus, and as an infant passage after dramatic changes during gestation and parturition. On the other hand, the cervix and mucus should prevent microorganisms in the lower reproductive tract which may induce pelvic inflammatory disease (PID). It is also the site of attack of specific sexually transmitted infection (STI) pathogens including *Chlamydia trachomatis* (CT), human papillomavirus (HPV), and human immunodeficiency virus (HIV).

A healthy cervicovaginal microbiota (CVM) and the acidic environment formed by the associated metabolites maintain cervical epithelial barrier integrity, stabilise the mucosal immune system, and balance host defence and tolerance with microbes, metabolites, components, and attachment to host cells. In contrast, microbiota dysbiosis and its accompanying changes in microbial metabolites induce an inflammatory response, cause damage to cervical cells, disrupt cervical epithelial and mucosal barriers, stimulate the immune system, and cause an imbalance in cervical homeostasis, thereby inducing various STI diseases. This review highlights recent advances in the understanding of the interactions between the microbiota and the cervical mucosal barrier (epithelial, mucus, and immune), and discusses the implications for cervical-specific STIs.

## Normal physiology state

### Anatomy and histology of the cervix

The cervix is a cylindrical structure approximately 2-cm long, with the cervical canal in the centre, the inner orifice opening to the uterus, and the outer opening to the vagina. The cervix is divided into the ectocervix and endocervix depending on whether it is exposed to the vagina. The cervix is composed of stroma and epithelial cells separated by a basement membrane. The underlying stroma of the cervix is predominately an extracellular matrix, including type I and type II collagen, and a small amount of type IV collagen (Leppert, 1995). Approximately 70% of the cervix’s extracellular matrix is composed of type I collagen, which plays a crucial role in maintaining tissue integrity (Yellon, 2019). Type IV collagen can be found in the basement membrane as well as enriched in the placenta (Lithgow et al., 2022). Other components included elastin, proteoglycans, hyaluronan, fibroblasts, and scattered immune cells (Tantengco et al., 2021a). Epithelial cells are region-specific, and the endocervix is covered by columnar epithelial cells. The ectocervix and vagina are covered by continuous stratified non-keratinised squamous epithelial cells. The area between the endocervix and ectocervix is the transformation zone, which is covered by squamous columnar epithelial cells. Intercellular junctions and the epithelial cells formed cervical epithelial barrier. The main connections between epithelial cells include tight junctions, adherent junctions, and desmosome junctions (Blaskewicz et al., 2011). Columnar epithelial cells are mainly composed of tight junctions that are directly regulated by oestrogens, cytokines, and growth factors (Grant and Wira, 2003; Wira et al., 2010). The squamous epithelium mainly consists of adhesive junctions and desmosome junctions, while tight junctions are lacking, allowing small molecules to pass through the intercellular space. Pathogenic microorganisms can contact Langerhans cells and CD4^+^ T cells distributed in the cervix (Hladik et al., 2007). In addition, epithelial cells can act as antigen-presenting cells (APCs) to recognise pathogenic microorganisms and secrete immune components such as antibiotics, cytokines, and chemokines.

### Cervical mucus barrier

Cervical mucus is produced by goblet/secretory cells around the crypts in the endocervical canal (Han et al., 2017) and contains mucins (MUCs), immunoglobulins, plasma proteins, lipids, sterols, carbohydrates, inorganic ions, and water. Mucin is a large O-linked glycosylated protein and is the main component of the cervical mucus (Radtke et al., 2012). Currently, 20 mucins are known, which are divided into gels such as MUC5AC, MUC5B, and MUC6, transmembrane proteins such as MUC1 and MUC16, and small soluble molecules (Andersch-Björkman et al., 2007; Radtke et al., 2012). MUC5B and MUC5AC are the main mucins of the cervix (Gipson et al., 1999; Gipson et al., 2001), and MUC5B is the main mucin that affects the properties of the cervical mucus. During the follicular phase, the amount of mucus increases with increasing oestrogen levels, and the mucus gradually becomes thinner. During ovulation, the mucus levels increase by 10–20 times, MUC5B expression reaches its highest level, and the secretion becomes watery, which facilitates the entry of sperm into the upper reproductive tract (Han et al., 2021). During the luteal phase, MUC5B expression decreases owing to the effect of progesterone, and the cervical mucus gradually thickens (Gipson et al., 2001). The levels of immune factors such as IgG/IgA, lactoferrin, interleukin-10 (IL-10), and antimicrobial peptides also show a bimodal distribution with the menstrual cycle (Ulcova-Gallova, 2010), with higher levels in the early stage of the follicular phase, followed by 10-1,000 times decreases in the middle of menstruation, and increases in the later stage of menstruation. However, the levels of IFN-γ, IL-4, IL-6, and IL-12 did not show periodic changes (White et al., 1997; Han et al., 2017). Hughes performed a meta-analysis include 31 article mainly CVL samples, found that antibodies, CC−type chemokines, MMPs, IL−6, IL−16, IL−1RA, G−CSF, GNLY, and ICAM1 were lower in the luteal phase than the follicular phase. IL−1α, HBD−2, and HBD−3 were elevated in the luteal phase. CXCL8, 9, and 10, interferons, TNF, SLPI, elafn, lysozyme, lactoferrin, and IL-1β, 2, 10,12, 13, and 17A show minimal change. Although some constituents showing cyclic variations increase the vulnerability of sperm passaging and pathogen ascent, some cytokines did not change with the cell cycle which may compensate for the risk of ascending infection (mucus increase) during ovaluation (De Tomasi et al., 2019).

The cervical mucus can play a barrier role in both physical and immunological aspects. First, mucin and other substances capture pathogenic microorganism particles and slow down the diffusion speed to gain time for local immune responses (Boukari et al., 2009; Shukair et al., 2013). Second, immune factors such as IgG, IgA, lactoferrin, and SLPI in cervical mucus can inhibit the adhesion and invasion of cervical epithelial cells by pathogenic microorganisms such as CT, and HIV (Radtke et al., 2012; Filardo et al., 2019).

### Distribution of local immune cells in the cervix

Studies on cervical immune cells mainly focus on cervical tissue and secretion samples. Neutrophils are the most prevalence immune cells found in cytobrush secretion samples, supported by studies from non-pregnant- (Mohammadi et al., 2020) and pregnant women (Hunter et al., 2016); this differs from samples collected from biopsies (Trifonova et al., 2014). Both cervical and vaginal tissues are mainly composed of APCs and T cells, and most of them are effector memory- or effector T cells, indicating that the cervical region is an organ dominated by cellular immunity (Trifonova et al., 2014). The content of APCs and T cells is higher in the cervix than those in the vagina, with the transformation zone and endocervix having the largest and smallest amounts, respectively (Pudney et al., 2005). In the ectocervix, 37–55% are APC cells (most are macrophages), followed by dendritic cells (DCs), and 23–43% are T lymphocytes. The number of APCs in the endocervix is similar to that in the ectocervix, however, T lymphocytes are approximately half of those in the ectocervix (Trifonova et al., 2014). CD8^+^ T cells are slightly more abundant than CD4^+^ T cells, with the latter accounting for approximately 50% of the total CD3^+^ cells in the endocervix and ectocervix. Th17 cells with CCR5 and CD90 co-expression are the main CD4^+^T cell population in the cervix (Rodriguez-Garcia et al., 2014).

Human cellular immunity works through the following processes: local APCs recognise pathogenic microorganisms, present antigens to lymphoid tissues, and T cells are stimulated and differentiate into effector T cells in local tissues. Subsequently, some effector T cells develop into memory T cells, which are divided into central memory T cells (TCM) that are stored in the extralymphatic tissue, and effector memory T cells (TEM) which can travel between the blood and extralymphatic tissue and play a role in circulating immune surveillance. Recent work found that some memory T cells also reside in local tissues and become tissue-resident memory T cells (TRMs) (Gebhardt et al., 2013; Sathaliyawala et al., 2013). An increasing number of studies confirmed that circulating memory cells fail to approach the genital tract mucosa at a steady state, while TRMs can quickly play a local immune role without being recirculated through lymphoid tissue (Iijima and Iwasaki, 2014). CD8^+^TRM (CD69^+^CD103^+^) is the predominant fraction in CD8^+^T cells of cervicovaginal tissue, while others are CD69^+^ single positive and double negative CD69^−^CD103^−^ cells, which are also defined as inflammatory mucosal T cells (Tim) (Pattacini et al., 2019). Most CD8^+^TRMs in the mouse reproductive tract express CD103, which binds to E-cadherin and promotes TRM retention within certain epithelial compartments (Rosato et al., 2017). Only a few CD4^+^TRMs express CD103, and CD4^+^TRMs are mainly distributed within memory lymphocyte clusters (MLCs). Once TRM recognises the cognate peptide, it releases cytokines IL-2, TNFα, and IFNγ, thereby upregulating adhesion molecules and chemokines and promoting the recruitment of circulating memory T cells and B cells into tissues (Schenkel et al., 2014; Rosato et al., 2017).

Studies on the effects of the menstrual cycle and menopause on cervical lymphocytes are scarce and controversial. Several studies found that the total number of CD8^+^T and CD4^+^T cells in the cervix does not change with the menstrual cycle and menopause (Pudney et al., 2005), and the toxicity of CD8^+^T cells is unaffected (Rodriguez-Garcia et al., 2020). In addition, endometrial CD8^+^T cells are inhibited by an increase in transforming growth factor beta (TGF-β) in the secretory phase, but cervical CD8^+^T cells are insensitive to TGF-β regulation. This means that the cervix can still play an immunoprotective role in preventing pathogenic microorganisms from ascending when the secretory endometrium is in an immunosuppressed state due to embryo implantation (Rodriguez-Garcia et al., 2020). However, other studies show that the menstrual cycle and age can affect the number and function of certain lymphocyte subsets. For example, the level of chemokine (C-C motif) ligand 2 CCL2 and the proportion of local CD4^+^TRM in the cervix increases during the follicular period (Boily-Larouche et al., 2019). CCL2 is a chemokine found in monocytes/macrophages, T cells, and TRM. The inhibitory effect of progesterone on CCL2 decreases during the follicular phase, and more CD4^+^TRMs are recruited to the local cervix (Hall and Klein, 2017; Boily-Larouche et al., 2019). In addition, the abundance of CD8^+^TRM and DC cells in the cervical tissue of postmenopausal women decreases with age (Rodriguez-Garcia et al., 2018). This may be the reason for the increased susceptibility of postmenopausal women to STI (Ghosh et al., 2014) and the second peak of HPV infection at the age of 45 (Rodriguez-Garcia et al., 2018).

### Normal cervicovaginal microbiome

The microbiota of the lower genital tract is identified more frequently than that of the endocervix. Using self-collected vaginal secretions samples, five “communities state types” (CSTs) are classified according to the composition and proportion of vaginal microbiota by Ravel et al. Specifically, CST-I, CST-II, CST-III, and CST-V corresponds to a predominance of *Lactobacillus crispatus, L. gasseri, L. iners*, and *L. jensenii*, respectively, whereas CST-IV is characterised by a combination of various facultative anaerobes with a low abundance of *Lactobacilli*. CST IV-A comprises *Anaerococcus, Prevotella, Corynebacterium, Peptoniphilus, Finegoldia*, and S*treptococcus*. CST IV-B is characterised by *Atopobium, Gardnerella, Mobiluncus, Sneathia, Megasphaera*, and other bcteria in the order *Clostridiales* (Ravel et al., 2011). In 2020, they comprehensively re-identified common vaginal CSTs in 1975 women with different ethnicities; black, white, Hispanic, and Asian. CST IV is divided into CST IV-A, IV-B, and IV-C. CST IV-A has a high level of *Candidatus Lachnocurva vaginae* (formerly called bacterial vaginosis-associated bacterium-1, BVAB1) and a moderate amount of *Gardnerella vaginalis*, whereas IV-B has a high abundance of *G. vaginalis* and a low amount of *Ca. L. vaginae*. Both IV-A and IV-B contain moderate levels of *Atopobium vaginae*. CST IV-C is characterised by other diverse anaerobic bacteria and is divided into five sub-CSTs: CST IV-C0 is a homogeneous community with a moderate level of *Prevotella* spp., CST IV-C1, C2, C4 with *Streptococcus* spp.*, Enterococcus* spp., *Staphylococcus spp*, as a dominated flora respectively. CST IV-C3 with *Bifidobacterium* spp. as dominant flora (France et al., 2020). In addition, “cervicotype” (CT) was proposed by Anathar et al. (Anahtar et al., 2015) by analysing the microbiota community structure of ectocervical swabs. CT1 is primarily dominated by non-iners *Lactobacillus*, CT2 is dominated by *L. iners*, CT3 by *Gardnerella*, and CT4 by diverse anaerobes associated with BV. Buck et al. further defined the structure of vaginal flora with “vagitype” (Fettweis et al., 2019). Although both vagitypes and dominant bacteria refer to bacterial species with a relative abundance of over 30%, each sample can have over two dominant bacterial species, and vagitypes refer to the most dominant and functional bacterial species. Normal vaginal flora is often composed of only one or two dominant bacteria due to the strong adhesion ability of *Lactobacillus*.

The microbiota in the vagina, endocervix, and uterine cavity of the same female show continuity. The overall microbiota biomass shows a downward trend and bacterial diversity gradually increases. The microbiota in the endocervix is more similar to the vagina than the uterus and is dominated by *Lactobacillus*. However, the proportion of *Lactobacillus* in the endocervix is lower than that in the vagina, and the relative abundance of *Bacteroides, Pseudomonas*, and *Prevotella* spp. increases. The relative abundance of *Lactobacillus* in the uterus is lower than in the vagina and endocervix (Chen et al., 2017; Zhang et al., 2021; Wang et al., 2021a). It is suggested that microflora quantity and composition is altered with the rise in genital tract position. The cervix is located between the vagina and uterus, and the characteristics of its microflora are between those of the vagina and uterus, suggesting that it is the transformation zone of bacteria in the reproductive tract.

CVM can be dynamic with the menstrual cycle and age. During menses, increased diversity (Krog et al., 2022) and decreased *Lactobacillus* abundances (Song et al., 2020) were observed. During the follicular and luteal phases, the abundance of *Lactobacillus* is expanded, and the vaginal microbiome of more women becomes dominated by *Lactobacillus* due to the increase of estradiol (Gajer et al., 2012; Song et al., 2020; Jie et al., 2021; Krog et al., 2022; Oerlemans et al., 2022). In postmenopausal women, species richness was decreased, but species diversity was significantly increased (Kim et al., 2021). The abundance of lactobacillus is decreased, while the proportion of anaerobic bacteria is increased (Gliniewicz et al., 2019; Kim et al., 2021). The composition of these communities resembles those of premenopausal women with BV and is correlated with symptoms of vaginal discomfort (Shardell et al., 2021; Mitchell et al., 2021a). However, the bacteria associated with BV nugent score in postmenopausal women were different from premenopausal women, which demonstrated there are differences in microbial community with premenopausal women with BV (Mitchell et al., 2021b).

Many studies also demonstrate that CVM is associated with disease treatment outcomes. For example, some studies reveal that low baseline *Lactobacillus* (Xiao et al., 2022), *L.iners*.(Zhou et al., 2022), high *G.vaginalis* and *A. vaginae* (Bradshaw et al., 2006; Ferreira et al., 2017) was associated with BV treatment failure and recurrence, although some reports show inconsistent effect. The discrepancy is partly due to different study designs, populations and technology. In addition, the different virulence of species (Mollin et al., 2022; Zhou et al., 2022), and the dynamics of the species in CVM (Xiao et al., 2019), rather than the genus as a whole maybe provide more information to monitor the treatment outcome.

## Interaction between microbiota and cervical barrier

### Microbiome dysbiosis and metabolite alterations

The microbiota and metabolites changed during cervicovaginal dysbiosis. *Lactobacilli* are the dominant species in healthy conditions and exclusively utilise sugars such as glycogen and glycogen hydrolysates as carbon and nitrogen sources to produce lactic acid (Amabebe and Anumba, 2018). *Lactobacilli* produce lactic acid, bacteriocins, and biosurfactants to protect the cervicovaginal environment. Bacterial vaginosis (BV) is one of the most common dysbiosis in the vagina, characterised by a decrease in *Lactobacillus* and an increase in anaerobic bacteria, such as *G. vaginalis, Atopobium* spp.*, and Prevotella* spp., which correspond to CST IV-A, IV-B, and IV-C0. Aerobic vaginitis (AV) is a vaginal dysbiosis characterised by the loss of *Lactobacillus* and an increase in aerobes such as *Enterococcus* spp.*, Escherichia coli, Staphylococcus* spp., and *Streptococcus* spp. identified by culture and bacterial sequencing (Wang et al., 2020), which may correspond to CST IV-C1, IV-C2, and IV-C4. A healthy metabolite environment positively correlates with the metabolism of lysolipids, phospholipids, glutathione, and glycogen, but negatively correlates with the metabolism of biogenic amines, lysine, and histidine, and is characterised by high lactic acid levels. Both *L. crispatus* and *L. gasseri* produce L-type and D-type lactic acids, *L. jensenii* only produces D-type lactate, and *L. iners* only produces L-type lactate (Witkin et al., 2013). *L. iners* also secretes the cholesterol-dependent cytotoxin, haemolysin. This explains why the dominant vaginal bacteria in healthy women are mostly *L. crispatus*, followed by *L. gasseri* and *L. jensenii*, while *L. iners* is often associated with pathogenic bacteria, for instance, *G. vaginalis*. Metabolites associated with BV positively correlate with the metabolism of biogenic amines, lysine, and histidine; however, they negatively correlate with lipid-, glutathione-, and glycogen metabolism. The levels of biogenic amines (putrescine, cadaverine and trimethylamine) and short-chain fatty acids (SCFAs) (especially acetate, butyrate and formate) in BV are high, while the levels of some amino acids (tyrosine and glutamate) in BV are low (Srinivasan et al., 2015; Vitali et al., 2015; Watson and Reid, 2018; Borgogna et al., 2020). The metabolite signature of AV is less characterised than that of BV. A study on premature rupture of membranes (PROM) patients shows that the glycolytic metabolite GalNAc (N-acetylgalactosamine) and sucrose negatively correlate with *Streptococcus, Chlamydia, Prevotella*, *Staphylococcus*, *Mycobacterium*, and *Enterobacter* genera, which supports the reduction of lactic acid (Liu et al., 2021b). In addition, *Streptococcus* spp. dominated communities contain slightly higher acetate levels (Gajer et al., 2012).

### Immune response induced by cervicovaginal microbiota on cervix

The effect of cervicovaginal bacteria on the cervix is less pronounced than on the vagina. Cervical epithelial cells and immune cells recognize and sense pathogens through pattern recognition receptors (PRRs). Cell-surface PRRs toll-like receptors (TLRs) 1,2,4,5,6 and 3,7,9 (Aflatoonian and Fazeli, 2008; Benjelloun et al., 2020) and intracellular cytosolic pathogen sensing receptors Nod-like receptors (NLRs), NOD1, NOD2, RIG-1, and MDA5 (Ghosh et al., 2013) have been found in the cervix mucosa. TLR1, TLR2, and TLR4-6 detect microbial ligands, TLR2 and TLR4 can recognize Gram-positive and Gram-negative bacteria, respectively, and regulate the release of downstream cytokines. NOD1 and NOD2 can recognize intracellular PAMPs that can enter the cell through phagocytosis or membrane pores (Anton et al., 2022). BV and associated pathogens, such as *Prevotella* and *Gardnerella*, have been shown that associated with the expressions of TLRs and NLRs, especially TLR2/4 (Anahtar et al., 2015; Anton et al., 2022; Dong et al., 2022; Gerson et al., 2022). BV-associated bacteria can induce immune response of cervical cells through the TLR2/4−activated signalling pathways (Zariffard et al., 2005; Anton et al., 2022). In addition, due to *G. vaginalis* can trigger the NLRP3 infammasome in macrophages and monocytes (Vick et al., 2014; Xiang et al., 2021), it is possible that NOD signalling may be another passway of G. vaginalis-mediated infammation in cervicovaginal epithelial cells (Anton et al., 2022).

Clinical studies of the relationship between cervical cytokines and CVM sequencing are listed in **Table 1**. Most studies show that the BV-associated microbiome (CST IV) and elevated microbiota diversity are related to an increase cervical IL-1α and IL-1β, and to a lesser extent granulocyte-macrophage colony-stimulating factor (GM-CSF) and IL-10 (Mitchell and Marrazzo, 2014; Shannon et al., 2017), which was similarly with vagina (Shannon et al., 2017). The *L. iners* dominant microbiome is associated with increased IP-10 and MIG compared with CST I/II (Anahtar et al., 2015; Shannon et al., 2017). Clinical data of IL-6, IL-8, and IL-10 cytokines and chemokines in the BV-associated microbiome are inconsistent in the vagina and cervix (Mitchell and Marrazzo, 2014). Discrepancies in immune molecules may be attributed to the diversity of microbes and hosts (Łaniewski and Herbst-Kralovetz, 2021). Several studies have explored the role of a single bacterium in epithelial immunometabolic characteristics using a 3D cervical epithelial model. Among the common vaginal bacteria, *Lactobacillus* (especially *L. crispatus*) usually plays a protective role in immunomodulation, *G. vaginalis* or *Prevotella bivia* usually induces a relatively low inflammatory response, whereas *A. vaginae* and *Sneathia amnii* elicit more robust cytokine responses (Eade et al., 2012; Gardner et al., 2020; Łaniewski and Herbst-Kralovetz, 2021). L. crispatus did not induce an inflammatory response or alter the epithelial barrier integrity when cocultured with 3D cervical models. P. bivia and G. vaginalis strains only induce IL-6, TNF-α, IL-1α, and MMP-9, whereas A. vaginae and S. amnii induce multiple proinflammatory molecules, including IL-6, IL-8, IP-10, MCP-1, MIP-3α, RANTES, MMP-10, MMP-1, and sFas. Polymicrobial infection with four BV-associated bacteria leads to a mixed profile and extra activation of IL-1β, a critical cytokine usually raised in women with BV (Łaniewski and Herbst-Kralovetz, 2021). Other BVAB such as Eggerthella sp. only causes an increase in IL-1α; Mobiluncus mulieris increases IL-1α, IL-6, IL-8, MCP-1, and TNF-α; while Megasphaera micronuciformis increases IL-1α, IL-1β, IL-1RA, TNF-α, IL-6, and sFasL (McKenzie et al., 2021). Cervical epithelial cells (especially endocervical epithelial cells) exhibit a more robust immune response than vaginal cells after stimulation by BVAB bacteria. The cervix and the transformation zone of the upper and lower reproductive tracts has a higher ability to generate inflammatory responses than that of the vagina, thus guaranteeing the relative sterility of the upper reproductive tract (Eade et al., 2012).

**Table 1:** The clinical studies of the relationship between cervical cytokines and CVM sequencing.

Author(year)	Population	Ethnicity	Sample	Method	Findings
Gustin et al, (2022)	BV N=28	NS	Vaginal swabs, CVL	16S V3-4	Shift from CT4 to CT2 possessing significantly higher levels of SLPI, GROa, and MIP3a and significantly lower levels of ICAM-1; Highly diverse microbiota are associated with the enhanced resilience of bacterial vaginosis to standard metronidazole treatment; CD4+ cells from the lamina propria were significantly higher in CT4
Lo´pez-Filloy et al, (2022)	Symptomatic cervical ectopy N=156	NS	Cervical secreations	16S V4	In HPV+ and cervical ectopy patients, bacterial diversity correlates with both IL-1βand IL-22 HPV+ women showed a significant decreased in *Lactobacilli* and increase in anaerobes such as *Sneathia, Pseudomonas, Megasphaera, Atopobium, Shuttleworthia, Prevotella*, and *Clostridium*, IL-21 and CXCL9 were significantly upregulated in HPV-positive
Kawahara et al., (2021)	CIN recieved surgery N=28 CIN under observation N=13	NS	Cervical secreations	16S V3-4	IL-1β and TNF-α were signifcantly increased with the presence of anaerobics microbiota; TNF-α, IL-10 and RANTES were inversely correlated with *L. crispatus*
Serebrenik et al., (2021)	Bronx N = 20 Thika N = 18	Mainly Black	CVL, Cervical tissue	16S V3-4 RNA-seq	BV patients who respond to metronidazole treatment had a increase of CXCL-9, CXCL-10, and SLPI and a decrease in IL-1α and IL-1β compared with treatment failure
Balle et al (2020)	130 females adolescent before and after contraceptive use	South African	Vaginal swab cervical secreations	16S V4	Cytokines were positely associated with the following aerobes. IL-17 (*S.anginosis,A.minutum, Mycoplasmataceae*), IL-6 (*peptococcus, Moryella*), IL-1b (*P.rnelaninogenia, Mycoplasma hominis,A.prevoii*), IL-21(*P.timonensis, P.rrica*), IL-23 (*S.sanguinus, Mycoplasmataceae*). The abundances of *L. crispatus* and *L. iners* were negatively correlated with the concentrations of several cytokines. The inflammation-high group had significantly higher alpha diversity compared to the low group and was more likely to be CST IV
Joag et al., (2019)	BV N=45	Keneya	Vaginal swabs, endocervical cytobrush	16S V3-4	BV treatment reduced genital CD4+ T-cell HIV susceptibility and reduced IL-1α/β levels, However, BV resolution and the concomitant colonization by *L. iners* substantially increased several genital chemokines associated with HIV acquisition, including IP-10, MIP-3α
Łaniewski et al. (2018)	HPV-negative controls N = 20, HPV-positive controls N= 31, Low-grade dysplasia N= 12, High-grade dysplasia N= 27 Invasive cervical carcinoma N = 10	Mix	Vaginal swab CVL	16S V4	Sneathia presence VMB associated with IL-1b, IL-36r, TNFa, IL-8,IL-10,MIP1a, TNFb,MIP-1b, RANTES, SCD40L,IL-10,

BV, bacterial vaginosis; CVL, cervicovaginal lavage; CST, community state types; KRST, Kenya, Rwanda, South Africa and Tanzania; CT, cerival type; CVM, cervicovaginal microbiota.

NS, Not specifically.

Research on common cervicovaginal bacteria in immune cells has mainly focused on DC cells, macrophages, and T cell recruitment and differentiation. The effect of *G. vaginalis* on DC cell stimulation and the inflammatory response is not obvious. *G. vaginalis* and its supernatants induce THP-1 macrophages to differentiate into the M1 phenotype which is involved in defence against bacterial infections, elevated reactive oxygen species (ROS) levels, and stimulation of the NF-κB/STAT1 pathway (Liu et al., 2021a). This causes THP-1 cell pyroptosis by promoting the formation of the NLRP3 inflammasome, resulting in cytokine secretion. Other BVABs such as *Megasphaera* elsdenii and Prevotella timonensis significantly promote the maturation of DC cells, the differentiation of T cells into the pro-inflammatory Th1 type, and the increase of IL-1β, IL-6, IL-8, IL-12p40, and TNF-α (van Teijlingen et al., 2020). The increase in Th1 pro-inflammatory cytokines Il-1β, IL-6, and IL-8 recruits Th1 and Th17 pro-inflammatory CD4^+^T cells, effector memory CD8^+^T cells, and leucocytes (Deruaz and Luster, 2015). In contrast, vaginal Lactobacillus inhibits the pro- or inflammatory response of epithelial or immune cells (Jan et al., 2012) and promotes M2 macrophages polarisation which is involved in tissue repair and wound healing (Lin et al., 2021), promotes the differentiation of CD4^+^T cells toward immunosuppressed Treg cells (Jang et al., 2017), but has no effect on DC maturation (van Teijlingen et al., 2020).

### Immune response induced by cervicovaginal metabolites on cervix

The most representative metabolites in the cervicovaginal tract are lactic acid and SCFAs. Lactic acid and other products such as extracellular polysaccharides (EPS) exhibit anti-inflammatory effects on both cervical epithelial and immune cells, while the effect of SCFAs is not well understood.

EPS secreted by vaginal L. gasseri strains G10 and H15 inhibit the inflammatory factor TNF-α and promote the increase in the anti-inflammatory factor IL-10 (Sungur et al., 2017). Coculture of protonated L/D lactate with cervical epithelial cells results in a decrease in IL-6 and IL-8, and an increase in IL-1RA and IL-1β (Hearps et al., 2017). IL-1RA is expressed more highly than IL-1β, and is an antagonist of IL-1β, which inhibits its pro-inflammatory effect (Hearps et al., 2017). Delgado-Diaz et al. (Delgado-Diaz et al., 2019) simulated the organic acid composition of healthy or BV women (normal: 33 mM L- lactic acid, 4 mM acetate, 1 mM succinate, pH 3.9; BV: 100 mM acetate, 20 mM succinate, 4 mM propionate, 4 mM butyrate, pH 7). Normal organic acid dampening TLR-elicited stimulation of IP-10 MIP-3α, IL-6 of cervicovaginal epithelial cells. Regarding the effect of lactic acid on immune cells, tumour-related studies demonstrate that lactic acid has an immunosuppressive role by inhibiting the differentiation of monocytes and T cells, activity of cytotoxic CD8^+^T cells (CTLs), maturation of DC cells, and promoting polarisation of macrophages towards the M2 type (Ma et al., 2020).

The effect of SCFAs is studied more fully in the gut than in the female reproductive tract. SCFAs act as an energy source and immune modulator of the intestinal cell. Most studies show that SCFAs (especially butyrate) restore intestinal barrier function in inflammatory conditions by exhibiting anti-inflammatory effects in intestinal mucosa and inducing tight junction protein expression (Parada et al., 2019), although some studies show contradictory results. For instance, SCFA (acetic, butyric, and propionic acids) cocultured with peripheral blood mononuclear cells (PBMCs) and neutrophils significantly enhance TLR2 and TLR7 and induces IL-8 and TNF-α in a time- and dose-dependent manner (Mirmonsef et al., 2012). Propionate and butyrate also increased in vitro transmigration of neutrophils (Vinolo et al., 2009). SCFAs induces both effector and regulatory T cells by suppressing histone deacetylases and regulating the mTOR-S6K pathway (Arpaia et al., 2013; Park et al., 2015). The difference depends on the type, concentration and pH of the SCFAs and cell type. In the female reproductive tract aspect, BV organic acids (especially acetic and butyric acids) enhance the secretion of TNF-α after TLR1/2/3 stimulation of cervicovaginal epithelial cells but inhibit the production of IL-6, RANTES, and IP-10 (Delgado-Diaz et al., 2019). The exact role of SCFA in the reproductive tract immune cells remains to be elucidated.

### The effect of cervicovaginal dysbiosis on epithelial barrier disruption

Clinical proteomic and transcriptional studies show that CVM is associated with the disruption of the cervical epithelial barrier (**Table 2**). Dysbiosis of vaginal flora may disrupt the epithelial barrier by inducing an inflammatory response.

**Table 2:** Clinical studies of cervicovaginal proteome and RNA-sequencing.

Author(year)	Population	Ethnicity	Sample	Method	Findings
Delgado-Diaz et al. (2022)	Nugent-BV N=56 Non-BV N=57	Black	Cervicovaginal swab	16s rRNA Host proteome	Women with L. iners dominated flora contain primarily bacterial L-LDH, women with L. crispatus-dominated microbiome contain both L- and D-LDH Between high L-LDH and low L-LDH, 31 of different proteins having epithelial barrier function related gene ontologies include tight junction protein 1, keratin 8, dermokine
Serebrenik et al. (2021)	BV N=38 before and after metronidazole treatment	NS	Vaginal swabs CVL Ectocervical tissue	16s V3-4 RNA-sequencing	Responders vs nonresponders: lower levels of CXCL9 in cervicovaginal lavage on day 0. Concentrations of CXCL9, CXCL10, and monocyte chemoattractant protein 1 increased signifcantly between day 0 and day 35
Farr Zuend et al. (2020)	Healthy pregnant N = 23 Non-pregnant N= 25	NS	CVL	Human and Microbial proteome	Microbial proteome :L-lactate dehydrogenase, Glyceraldehyde-3-phophospate dehydrogenase type I, pyruvate kinase, and phosphoglycerate kinase were primarily driving the variance within the LD group, while transaldolase was responsible for the variation in the nLD group
Ferreira et al. (2018)	BV N=29 Health controls N=29	MIX	CVL	Human proteome	BV CVL: Neutrophil elastase, kaliocin-1, neutrophil defensin-1, Ig lambda-2 chain C regions, and protein S100-A7↑. Normal CVL: immune modulation and epidermis development leukocyte elastase inhibitor, serpin B4, cluster of proline-rich protein 2A, smallproline-richprotein3, cluster of cornifin-B, cellular retinoic acid binding protein 2, and fatty acid binding protein↑.
Bradley et al. (2018)	Lactobacilli dominant group N=32 Non-Lactobacilli dominant group N=15	NS	Cervicovaginal swab sampled at different menstrual cycle phase	16s V3-4 Human proteome	The luteal and follicular phases associate with higher activation of neutrophil/leukocyte pathways and cell migration pathways, Ovulatory phase exhibits increased of antimicrobial and wound-healing pathways and reduced inflammatory cytokines. Women without Lactobacillus showed an amplification of hormone associated variation such as the decrease in the epithelial barrier protein RPTN during the luteal phase
Borgdorff et al. (2016)	L. crispatus-dominated (group1) N=7 L. iners-dominated (group2) N=11 Moderate dysbiosis (group3) N=14 Severe dysbiosis (group 4) N=18	NS	Cervical spatulas and cytobrushes, CVL	Human proteome	From group1 to group4: AMP and cytokines(cystatin A,lysozyme C, ubiquitin ↓;histones, psoriasin, calprotectin↑, IL-36a, C5, MIF↑ Mucus (MUC5B、MUC5AC↑) Cytoskeleton alterations(KRT4、5、6A↓; LDHA, LDHB↑) Humoral immune response(IGHG1 and IGHG2↓)
Zevin et al. (2016)	cohort 1 n=10 coehort 2 n=31	Black(n = 27) Caucasian (n = 3) Asian (n = 2) Hispanic (n = 1).	Vaginal swabs CVL	16s V3-V4 Human and microbial proteome	Gardnerella vaginalis dominat women associated with host epithelial barrier disruption and enhanced immune activation; Lactobacillus species dominat women experised host mucosal proteins important for maintaining epithelial integrity.
Arnold et al. (2016)	Elevated inflammatory cytokines N=28 Controls N=68	Black	Endocervical cytobrush, CVL	Human proteome	Elevated inflammatory cytokines were associated with proteases, cell motility, and actin cytoskeletal pathways, whereas protease inhibitor, epidermal cell differentiation, and cornified envelope pathways were decreased.
Anahtar et al. (2015)	CT1 N=8 CT2 N=27 CT3 N=28 CT4 N=31	Black	Ectocervical vaginal swabs, CVL, endocervical cytobrush	16S V4 Whole-genome shotgun sequencing RNA-seq	The genes upregulated in APC of CT4 were enriched in NF-kB, Toll-like receptor (TLR), NOD-like receptor, TNF-a signaling pathways and many pro-inflammatory cytokine genes compared with CT1 and CT2
Cruciani et al. (2013)	Health N=40 BV N=39	NS	CVL	Human proteome	BV vs. Healthy: The majority of immunoglobulins↑, mucus (Mucin-5B↑) Epidermis development and keratinization (cadherin-1, Desmocollin-2, Desmoglein-3↑; Keratin5↓) Cytoskeleton remodelling, blood coagulation and complement activation were the most enriched passways

BV, bacterial vaginosis; CVL, cervicovaginal larvage; CT, cerival type. NS, Not specifically. ↑, increase; ↓, decrease.

Borgdorff et al. divided vaginal microbiota composition into four groups according to bacterial diversity: group 1 (*L. crispatus*-dominated), group 2 (*L. iners*-dominated), group 3 (moderate dysbiosis, mixture of BV), and group 4 (severe dysbiosis, all BV-positive). An increase in the vaginal microbiota group causes proteomic changes including cytoskeleton alterations (increasing actin-organising proteins, decreasing keratins and cornified envelope proteins), increasing lactate dehydrogenase (LDH) A/B as markers of cell death, proinflammatory cytokines, and proteolytic activity, together with decreasing immunoglobulin G1/2, AMP imbalances, and mucus alterations (increasing MUC5B and 5AC) (Borgdorff et al., 2016). Additionally, the cervicovaginal microbiome influences certain menstrual cycle-dependent changes to the cervical epithelial barrier, where hormone-associated reduction of the epithelial barrier protein RPTN is amplified in women without *Lactobacilli* during the luteal phase (Bradley et al., 2018). Other proteome studies demonstrate that CST III/IV or BV have differentially expressed proteins involved in the cytoskeleton, keratinisation alteration, epidermis development, and immune response compared with healthy women (Cruciani et al., 2013; Zevin et al., 2016; Ferreira et al., 2018). The inflammatory response contributes to the disruption of the cervicovaginal epithelium. Arnold et al. found elevated cytokine expression is positively associated with neutrophil proteases (MMP-9 and MMP-8), reduced antiproteases (SPINK5, SPINK7, SLPI), and altered cytoskeleton, epithelial differentiation, and keratinisation pathways. IL-1β, MIP-3α, and IL-8 display the strongest correlations with MMP-8 and MMP-9, indicating reduced barrier integrity (Mohammadi et al., 2022). IL-1β activates p38 and JNK signalling, leading to decreased tight junctions and disruption of mammary epithelial intergrity (Kobayashi et al., 2021). TNF-α and lipopolysaccharide (LPS) increase apoptosis, necrosis, and senescence of cervical epithelial cells (Tantengco et al., 2021a). The dysbiotic microbiome induces an inflammatory response which contributes to the disruption of cervicovaginal epithelial cells.

In addition, CVM and its metabolites directly influence the epithelial barrier by inducing oxidative stress, altering miRNA expression, and promoting cell cycle arrest and apoptosis. BV-associated bacteria induce oxygen stress intermediates when cocultured with a 3D cervical epithelial model which affects the epithelial barrier integrity (Wang et al., 2021b). *P. bivia* and *S. amnii* increase 2-hydroxyglutarate, 2-hydroxybutyrate, and citrulline levels, which are correlated to the activity of inducible nitric oxide synthase (iNOS) (Łaniewski and Herbst-Kralovetz, 2021). *Eggerthella* sp. and *M. mulieris* elevate sphingolipids and 2-hydroxybutyrate and decrease cysteinylglycine and cysteinylglycine disulphide (McKenzie et al., 2021). *Veillonella atypica* and *Veillonella montpellierensis* significantly accumulate histamine. *Fusobacterium gonidiaformans* and *Fusobacterium nucleatum* increase 2-hydroxyglutarate, induce cysteine- and methionine metabolic pathways, pro-inflammatory lipids, and genotoxic hydrogen sulfide (Maarsingh et al., 2022). 2-hydroxyglutarate is a metabolite marker of oxidative stress. The depletion of two intermediates in the glutathione synthesis pathway (cysteinylglycine and cysteinylglycine disulfide) indicates increased glutathione biosynthesis and an increase in ROS levels (McKenzie et al., 2021). The distribution of ROS or oxidative stress pathways in human cervical epithelial cells and cervical stromal cells is linked to sterile inflammation (increased IL-6). This is mediated by p38 mitogen-activated protein kinase activation which promotes cell cycle arrest and cell necrosis death (Tantengco et al., 2021b), and is associated with lipid peroxidation and epithelial tight junction disruptions (Yamamoto et al., 2021). Histamine decreases tight junctions (ZO-1 in nasal epithelia and E-cadherin in pulmonary epithelia) (Salliss et al., 2021). The increase in sphingolipids also reflects disruption of the epithelial barrier.

**Figure 1:**
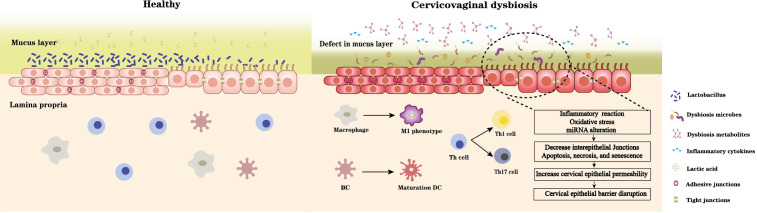
A healthy Lactobacillus dominant microbiota and the acidic environment formed by the associated metabolites especially lactic acid maintain cervical epithelial barrier integrity, stabilise the mucosal immune system. In contrast, microbiota dysbiosis and its accompanying changes in microbial metabolites can 1) damage mucus layer; 2) imbalance immune system: promote immune cells differentiate towards proinflammatory type and induce pro- and inflammatory cytokines secretion; 3) disrupt cervical epithelial barrier: induce inflammation reaction, oxidative stress, miRNA alteration of cervical epithelial cell, decrease intracellular junctions, and promote cell cycle arrest, apoptosis, and necrosis. Thus, increase the epithelial permeability and disrupt barrier function.

BV-associated bacterial supernatants also affect the alteration of epithelial barrier function. Supernatants derived from *G. vaginalis* and *L. iners* increase ectocervical and endocervical cell permeability, cleave E-cadherin, and elevate miR-143 and miR-145 expression, which downregulate cell adhesion genes, JAM-A and FSCN1, decrease cell proliferation, and increase apoptosis (Anton et al., 2017). *M. mulieris* supernatant also increases cell permeability (Dude et al., 2020). In contrast, lactic acid increase the expression of barrier proteins claudin1, claudin4 of cervicovaginal epithelial cells (Delgado-Diaz et al., 2022), *L. crispatus* supernatants also can mitigate the disruption of cervical epithelial barrier induced by LPS or *G. vaginali*s and reversal of *G. vaginalis*-induced increase in miRNA expression (Anton et al., 2018). *G. vaginalis* secretes haemolysin (vaginolysin), a cholesterol-dependent pore-forming toxin that increases the membrane permeability of host cells and causes K^+^ efflux, activates caspase-1, and contributes to apoptosis (Muñoz-Planillo et al., 2013; Vick et al., 2014). The increase of sFasL (an apoptosis-related protein) in cervicovaginal lavages of women with vaginal dysbiosis demonstrates the damage and apoptosis of cervicovaginal epithelial cells. Taken together, vaginal dysbiosis bacteria can directly induce epithelial barrier disruption through oxidative stress and miRNA alteration, and promote cell cycle arrest, apoptosis, and necrosis. An indirect effect is also realized by the secretion of harmful metabolites and causing immune disorders (**Figure 1**).

### The effect of cervicovaginal microbiota on mucus and cervical remodeling

Mucus and mucins play a role in protecting mucosal surfaces, protecting the epithelium from contact, and removing intruders by trapping (Hansson, 2020). Mucins provide an attachment site and a nutrient source for some bacteria. For example, isolated vaginal *Lactobacilli* have shown the ability to bind mucin (Leccese Terraf et al., 2014; Ahire et al., 2021). Vaginal *Lactobacilli* contain genes encoding proteins that allow adhesion to mucins, including mucus adhesion promoting protein (MapA), mucus-binding protein (MubI), cell and mucus-binding protein (CmbA), modulator of adhesion and biofilm formation (mabA), and mucus binding factor (MBF) (Leccese Terraf et al., 2014). *Lactobacilli* isolated from the gut contain several mucus adhesion proteins, including mucus-binding protein (MUB), MucBP domain, pili, SIpa, MapA, and EF-Tu. These adhesion proteins include two main patterns: MUB, the MucBP domain, and pili are cell wall-anchored proteins that covalently bond to the cell wall; MapA, EF-Tu, and ATP-binding cassette (ABC) transporters are multifunctional proteins (so-called moonlighting proteins) that, in addition to performing their primary intracellular functions, also act as adhesion molecules (Nishiyama et al., 2016).

BVAB alters the cervical mucus by stimulating mucin production and degrading highly glycosylated proteins. Clinical studies show that the levels of MUC1, MUC4, MUC5AC, MUC5B, and MUC7 are significantly higher in women with BV than in women with normal or intermediate Nugent scores (Borgdorff et al., 2016; Moncla et al., 2016). Bacterial and viral products and TNF-α increase the mRNA expression of MUC1, MUC4, and MUC16 in a 3D endocervical model (Radtke et al., 2012). An increase in MUC expression enhances the ability of epithelia to form a protective barrier, thus promoting bacterial clearance. Sialidase and glycosulfatase levels and activity increase in BV patients compared with normal women (Gipson et al., 1999; Gipson et al., 2001; Wiggins et al., 2002; Ng et al., 2021). *G. vaginalis* strains, *Prevotella* species, and *S. amnii* liberate or consume sialic acids (Łaniewski and Herbst-Kralovetz, 2021). Three sialidases are identified in *G. vaginalis* strains: NanH1 (formerly sialidase A), NanH2, and NanH3. Among these, NanH2 and NanH3 have higher ability against sialic acid substrates in many different molecular contexts, such as 2-3- and 2-6-linked sialic acids, and N- and O-linked sialoglycans found on SIgA and mucin (Robinson et al., 2019). The only Prevotella spp. that contains a gene encoding α-N-acetylglucosaminidase (an enzyme that cleaves O-glycans of type 3 mucins) is P*. timonensis*; consequently, this species has the greatest sialidase activity in this genus (Ilhan et al., 2020).

Vaginal dysbiosis is associated with cervical shortening (Di Paola et al., 2020) and PTB (Gerson et al., 2022). The cervix needs to be extensively remodelled during pregnancy to allow a full term foetus to pass through the birth canal, which is divided into softening, ripening, dilation/labour, and postpartum repair. Both the cervical epithelium and stroma undergo changes during this process. Cervical epithelia proliferate in preparation for parturition and regulate the expression of aquaporin water channels to maintain fluid balance, epithelial permeability, and a protective barrier. This regulates solute transport through the intracellular junctional complex and protects the cervical stroma and upper reproductive tract against invasion of pathogens (Timmons et al., 2007). Stromal changes include increasing hyaluronan content and collagen solubility (Read et al., 2007), and loosening of the collagen matrix (Yellon, 2019).

The potential mechanisms by which bacterial infections affect cervical remodelling mainly involve metalloproteinases. *G. vaginalis*, *A. vaginae*, *P. bivia*, and *P. asaccharolytica* can induce or secrete MMP-1, 9 and 10, which shows the ability to degrade collagen (type I and IV), gelatin, casein, and fibrinogen (Łaniewski and Herbst-Kralovetz, 2021; McGregor et al., 1986; Łaniewski and Herbst-Kralovetz, 2021; Short et al., 2021; Tantengco et al., 2021a), while *L. crispatus* has no effect on type I collagen, casein, and fibrinogen, which inhibits clot formation (Lithgow et al., 2022). The colonisation of the mouse reproductive tract by *G. vaginalis* increases mucin expression, dispersion of collagen fibres, and alters cervical biomechanical properties which indicates a more rapid cervical remodeling (Sierra et al., 2018). The ability to secrete or induce metalloproteases and degrade collagen highlights their potential to alter reproductive tissue structure and harm human pregnancy through premature cervical remodelling, clotting disruption, and foetal membrane weakening.

## The effect of cervicovaginal microbiota on STI infection

### CT infection

*Chlamydia trachomatis* (CT) is the most prevalent sexually transmitted bacterial infection worldwide, with approximately 131 million new cases occurring annually (Kreisel et al., 2021). In some cases, it ascends from the cervix to the uterus and fallopian tubes, leading to severe reproductive pathology (Brunham and Rekart, 2009). It is characterised by an asymptomatic infection and a high reinfection rate. The transmission rates of CT are between 25–40%. Only 50% of women will naturally resolve the CT infection within one year (Morré et al., 2002; Molano et al., 2005). CVM and its products may serve as important cofactors for infection susceptibility, clearance, or reinfection.

BV is the most frequent vaginal infection and is found in 20–48% of CT-infected women (Wiesenfeld et al., 2003; Filardo et al., 2019). A growing number of sequencing studies show the association of cervicovaginal microbiota with CT infection (**Table 3**). A recent meta-analysis reveals a trend towards a positive association between low *Lactobacilli* microbiota and HPV and CT infection, suggesting a potential protective role of high *Lactobacilli* microbiota (Tamarelle et al., 2019). *Lactobacilli* including *L. crispatus*, *L. gasseri*, *L. jensenii, L. reuteri*, and *L. aviarius* are reduced in both the cervical and vaginal microbiota of CT-positive women (Filardo et al., 2017). Asymptomatic CT-positive patients are more likely to have *L. crispatus* than women with CT-correlated symptoms. In contrast, BVAB, *L. iners*, and increased species richness and diversity are associated with CT infections (Filardo et al., 2017). A specific bacterial network characterised by BV-associated bacteria include, *G. vaginalis, Prevotella amnii, Prevotella buccalis, P. timonensis, Aerococcus christensenii*, and *Vatica guangxiensis* is a potential biomarker of CT (Filardo et al., 2019). In addition, *L. iners*-dominated CVM is an independent risk factor for CT infection compared to *L. crispatus*-dominant CVM (van der Veer et al., 2017; van Houdt et al., 2018).

**Table 3:** The clinical studies of cervicovaginal microbiota sequencing in CT infection women.

Author(year)	Population	Ethnicity	Sample	Method	Findings
Chen et al. (2021)	Tubal infertile women with CT(CT-P) N=9, without CT(CT-N) N=8 Healthy women without CT (CT-C) N=7	Asian	Cervical swabs	16S V3-V4	CT-P: The abundances of most *Lactobacillus*, including *L. crispatus, L. gasseri, L. jensenii, L. reuteri*, and *L. aviaries*↓; *L. iners* and *Veillonellaceae bacterium KA00182*↑; IFN-g and IL-10↓
Mott et al. (2021)	CT naturally cleared population N=13 CT persisted population N=42	Mainly black	Cervical cytobrush	16S V4	Vaginal microbiota may be a stronger predictor of genital cytokine signatures, which may more accurately explain differences in CT clearance. IFN-g *in vivo* were significantly below those needed to clear CT infections in *in vitro* models, also were not significantly different in CT clearers and non-clearers. IFN-g may act together with other factors *in vivo*.
Chiu et al. (2021)	CT positive patients N=22 Healthy controls N= 36	Asian	Vaginal swabs	16S V3–V4	In CT-positive patients, the vaginal microbiota was dominated by *L. iners*, and the relative abundance of *Gardnerella vaginalis* (12.46%) was also higher than that in TV-positive and healthy controls
Raimondi et al. (2021)	CT positive women N=10 Healthy controls N=16	Caucasian	Vaginal and anal swabs	16S V3–V4	In CT-positive patients, the abandance of *Lactobacilli* were reduced and with higher evenness; dysbiosis-associated bacteria (e.g., *Sneathia, Parvimonas, Megasphaera, Ezakiella* spp) were increased
Tamarelle et al. (2020)	CT positive women N=149 Healthy controls N=99 Test every 3 months, a total 3 times	Mainly black	Vaginal swabs	16S V3–V4	CT+ women: was dominated by *Lactobacillus iners*(CST III) or a diverse of bacterial vaginosis–associated bacteria (CST IV). *L. iners*–dominated communities were most common after azithromycin treatment, suggesting that the impact of antibiotic treatment on the vaginal microbiota could favor reinfections.
Filardo et al. (2019)	CT positive women N=42 Healthy controls N=103	NS	Cervical swabs	16S V4	CT+ women: cervical microbiota categorized CST-IV were dominant. A specific bacterial network was identified as a potential biomarker, characterized by *G.vaginalis, P. amnii, P. buccalis, P. timonensis, A. christensenii*, and *V. guangxiensis*. Cervical lactoferrin, IL-6, IL-1, IFN-β,and IFN- α ↑, IFN- γ↓
Cheong et al. (2019)	CT positive women N=40 Healthy controls N=30	Asian	Cervical swabs	16S V3–V4	CT+ women: mostly strict and facultative anaerobes such as *Streptococcus, Megasphaera, Prevotella, and Veillonella genera*↑
Parolin et al. (2018)	CT positive women N=20, Healthy controls N=22 BV N=30	Caucasian	Vaginal swabs	Vaginal Array	CT+ women: *L. iners*↑, Asymptomatic CT-positive patients are more likely to have *L. crispatus* than women with CT-correlated symptoms, 4-aminobutyrate were lower in asymptomatic than symptomatic CT women
Balle et al. (2018)	CT positive women N=30 Healthy controls N=42	NS	Vaginal and cervical swabs	16S V4	CT+ women: more likely to have community type dominated by diverse anaerobic bacteria (C1) or L. iners (C3)
Di Pietro et al. (2018)	HPV/CT positive women N=30 Healthy controls N=43				HPV/CT co-infected women had a higher microbial diversity, Aerococcus christensenii was associated with C. trachomatis infection.
Filardo et al. (2017)	CT positive women N=10 Healthy controls N=35	NS	Cervical swabs	16S V3–V4	CT+ women: an overall decrease in Lactobacillus spp, diversity and anaerobes ↑
van der Veer et al. (2017)	CT positive women N=52, Healthy controls N=41	Mainly European	Cervical and/or vaginal swab	16S V3–V4	Diverse anaerobic CVM and L. iners–dominated CVM were independently associated with CT infection compared with women with L. crispatus–dominated CVM

CT, Chlamydia trachomatis. NS, Not specifically. ↑, increase; ↓, decrease.

Some studies have explored the mechanisms of CVM in CT treatment and clearance. Mott et al. (Mott et al., 2021) evaluated the potential impact of BV and metronidazole treatment on CT clearance and found that CT clearers were more likely to have a *Lactobacillus*-dominant vaginal microbiota after metronidazole treatment compared to non-clearers. The diverse anaerobe dominant group has higher IL-1α and IL-1β levels, while the *L. crispatus* or *L. iners* dominant group has higher IL-6 and IP-10 levels, which is consistent with CT clearers. IP-10 can result in a rapid influx of T cells which may aid CT clearance. IP-10 can be abrogated in endocervical epithelial cell secretion after CT infection. Cervicovaginal dysbiosis and BV treatment can drive cytokine changes, which may modulate CT clearance. Although *L. iners* dominant flora and its relative cytokine IP10 may help CT clearance. *L. iners* and *G. vaginalis* have a higher level of resistance to azithromycin and, thus could be selected for post-treatment, whereas sensitive *Lactobacillus* spp. are diminished (Tamarelle et al., 2020). The instability of the *L. iners* dominant flora, its ease of conversion to BV-associated flora, and the potential risk factors for CT infection suggest that the risk of STI in women may not be reduced after antibiotic treatment.

IFN-γ induced persistence of CT in epithelial cells is another issue through which CVM can impact the pathogenesis of CT. CT is characterized by two distinct forms, including extracellular infectious basic bodies (EBs) and intracellular dividing reticulums (RBs). CT is a tryptophan auxotroph. IFN-γ produced by infiltrating T and NK cells induces the activation of the enzyme IDO1, which depletes the tryptophan into kynurenine. Lacking tryptophan can arrest CT in intracellular RBs, resulting in morphologically aberrant, viable but non-cultivable, persistent growth form (Beatty et al., 1994; Aiyar et al., 2014; Ziklo et al., 2019). CT contain a tryptophan synthase operon (trpA, trpB and trpR genes) that can produce tryptophan from indole. *Lactobacillus* spp. do not produce indole, however, many *Prevotella* spp. can produce indole and promotes CT infection in the presence of IFN-*γ* (Ziklo et al., 2016). Besides *Prevotella* spp.*, Porphyromonas asaccharolytica, Propionibacterium acnes, Fusobacterium nucleatum, Faecalibacterium prausnitzii, Enterococcus faecalis, Peptoniphilus harei*, and *Escherichia coli* are also found the ability of indole-producing (Ziklo et al., 2018). In addition to tryptophan depletion, a recent study found that IFN-γ also can down regulate c-Myc, the key regulator of host cell metabolism, resulting in the reduction of TCA cycle intermediates and nucleotides to prevent chlamydial replication and promoting persistence (Rajeeve et al., 2020; Vollmuth et al., 2022). Constitutive expression of c-Myc can rescue CT from persistence induced by IFN-γ. Dong et al. (Dong et al., 2022) proved that the abundance of *Prevotella* correlates with the expression of cervical TLR4, NF‐κB, C‐myc, and hTERT genes. In contrast, *Lactobacillus* abundance showed negative correlations with these genes. Whether CVM can impact IFN-γ involved CT pathogenesis and persistence through C-Myc remain to be verified.

Most experimental studies have concentrated on the protective effect of *Lactobacillus* spp. towards CT infections. CT replicates only intracellularly; therefore, attachment and entry are essential in CT infection and pathogenesis. (Mastromarino et al., 2014). *L. brevis* and *L. salivarius* can co-aggregate with CT and compete with HeLa cells, thereby reducing the proportion of EB and inhibiting EB absorption. The intracellular multiplication of CT are inhibited by *L. brevis* and *L. salivarius* (independent of the pH) (Mastromarino et al., 2014). Vaginal *Lactobacilli* inhibit CT adhesion and infectivity in human epithelial cells and macrophages (Rizzo et al., 2015; Parolin et al., 2018). Incubation of HeLa cells with *L. crispatus* BC5 cells reduces polar lipids and α5β1 integrin subunit exposure in the epithelial plasma membrane. The interaction of CT with the β1 integrin subunit of host epithelial cells is a mechanism for EB binding, invasion, and signalling (Stallmann and Hegemann, 2016).

*Lactobacillus* supernatants inhibit CT infectivity; *L. crispatus* is the most potent while *L. iners* is the least effective (Edwards et al., 2019; Chen et al., 2021). The supernatants of D-lactate-producing Lactobacillus spp.(*L. crispatus* and *L. jensenii*) modulate the expression of multiple genes related to cell proliferation including decreasing miR-193b and histone deacetylase 4 (HDAC4) which are required for CT-induced proliferation during infection. HDACs can repress gene transcription of cyclin-dependent kinase inhibitor 1A (CDKN1A), which in turn inhibits the activity of cyclin-dependent kinase 4 (CDK4) and thus the cell cycle. Downregulation of HDAC4 by *L. crispatus* and *L. jensenii* supernatants led to an increase of CDKN1A and lower host cell proliferation. Therefore, these extracts help reduce susceptibility to CT infection (Edwards et al., 2019). D-lactic acid is the most potent metabolite and could compensate for the inhibitory effect of *L. iners* on CT detection. Lactoferrin is another metabolite that inhibits CT entry into host cells and downregulates IL-6/IL-8 synthesis. Its inhibitory role is proven by the administration to pregnant women asymptomatically infected with CT (Sessa et al., 2017). *Lactobacilli* and their products also modulate the host inflammatory reaction after CT infection. For instance, *L. crispatus* and its supernatant reduce production of IL-6, IL-8, and TNF-α and increase IL-10 production in CT-infected HeLa and J774 cells (Rizzo et al., 2015). *Lactobacilli* mixtures significantly reduce CT-induced cytokine production (TNF-α, IFN-γ, and IL-1β) in the mouse vagina and the severity of hydrosalpinx, oviduct inflammation, and dilatation (Chen et al., 2022). *L. crispatus* reduces the levels of pro-inflammatory cytokines to reduce inflammatory symptoms, which may explain why asymptomatic CT-positive patients are more likely to have *L. crispatus* than women with CT-correlated symptoms.

### HPV infection and relative disease

Most existing epidemiological studies support a positive association between vaginal infection and HPV acquisition, persistence, and cervical intraepithelial neoplasia (CIN) progression. Many case-control-, cross-sectional-, longitudinal-, and meta-analysis studies demonstrate that BV is the most relevant vaginal infection. A meta-analysis including 13 studies demonstrated that the factors related to HPV infection are BV, vulvovaginal candidiasis (VVC), CT, and ureaplasma urealyticum (UU), with BV also related to CIN. Another study also showed that moderate-to-severe AV are correlated with CIN (Plisko et al., 2021). A network meta-analysis of the microbiome including 11 longitudinal and cross-sectional studies showed that a low *Lactobacilli*-dominated CST and greater microbiota diversity are strongly associated with HPV compared with *L. crispatus*. *L. iners* also showed higher odds of HPV infection than *L. crispatus* (Norenhag et al., 2020; Usyk et al., 2020; Zhang et al., 2022). Women who acquire HPV16 are more likely to transition between microbial communities than women without a previous history of HPV infection (Moscicki et al., 2020). Taken together, vaginal dysbiosis associated with CST, higher diversity, and unstable transition properties are associated with HPV infection and progression (**Supplementary Table 1**). Most studies have shown the harmful role of BVAB in HPV infection and related diseases, including *Gardnerella, Prevotella, Atopobium*, *Sneathia*, and *Mycoplasma*, although a few studies show inconsistent results, which is the same as that of *L. iners*. Another longitudinal cohort study found that depletion of *Lactobacillus* spp. and the existence of specific anaerobic bacteria (including *Megasphaera* spp.*, P. timonensis and G. vaginalis*) are associated with persistence and slower regression of CIN2 (Mitra et al., 2020).

As previously discussed, BV-associated bacteria can induce cervical inflammation, influence local immunity, and disrupt the epithelial barrier. This impact may contribute to HPV infection and disease progression. HPV infection also promotes cervicovaginal microbiota imbalance by influencing the expression of mucosal host defence peptides. Lebeau et al. demonstrated that HPV E7 oncoprotein substantially inhibits host defence peptide expression including HβD1, 2, 4, HD-5/6, SLPI, S100A7, and elafin by interacting with NF-κB and Wnt/β-catenin signalling pathways. All these peptides have antimicrobial activity against *G. vaginalis* except HβD1. Meanwhile, S100A7 and elafins expressed by the cervical/vaginal squamous mucosa can be cleaved, internalised, and used as an amino acid source by *Lactobacilli*, which leads to its survival. Consequently, the amino acid source supporting the survival of *Lactobacillus* species is considerably decreased, promoting an unbalanced cervicovaginal flora (Lebeau et al., 2022). This is consistent with work showing that HPV infections result in an increase in vaginal bacterial richness and diversity and a decrease in the percentage of *Lactobacilli*; this causes a shift from CST III to CST IV, despite the status of CINs (Chen et al., 2020).

CVM can affect HPV infection by modulating the inflammatory environment. The antiviral-specific immune response to HPV infection needs the cooperation of CD4^+^T helper cells and cytotoxic CD8^+^T cells (CTLs). High levels of IFN-γ secreted by Th1 cells potentiate the cytotoxic activity of CTLs and target pathogen-infected cells. IFN-γ also promotes the expression of intracellular antiviral genes that block viral replication (Hickey et al., 2011). In contrast, Th17 and IL-17 are associated with an enhanced cervical immune response during HPV infection, result in the progression of cervical lesions (Sahu and Khare, 2021). The number of Th17 cells in the blood and the level of IL-17 in tissue homogenates of the cervix increases at different stages of cervical lesions (Xue et al., 2018). Nicolò et al. stimulated SiHa and CaSki cells with heat-inactivated bacteria to explore the relevant changes in cytokines: Lactobacilli (especially *L. gasseri* and *L. jensenii*) significantly induce IFN-γ levels, while L. ines and *G. vaginalis* increase IL-17 in addition to IFN-γ (Nicolò et al., 2021). An increase in IL-17 and IL-17-inducing cytokines (IL-23 and IL-1β) are observed in the cervicovaginal lavage obtained from women with CST-IV-dominated microbiota (Gosmann et al., 2017). Besides *G. vaginalis*, *Streptococcus anginosus*, *Staphylococcus*, and *Mycoplasmataceae* correlated with an increase in IL-17, cervical lesions, and cancer (Fan et al., 2021b; Manzanares-Leal et al., 2022). In contrast, dominant flora composed of *L. gasseri* and *L. jensenii* are associated with HPV clearance and the prevention of low-grade cervical dysplasia progression (Brotman et al., 2014; Mitra et al., 2015). In addition to IL-17, continuous low IL-1β/IP-10 (BV cytokine signature), predicts HR-HPV clearance, and an elevated TNF-α/MIP-1β signature is associated with CIN2+ progression (Usyk et al., 2022).

Regarding the role of cervicovaginal organic acids in HPV and related diseases, many experts have explored whether *Lactobacilli* and their suppernant have a protective effect against disease. *L. crispatus* culture supernatants promote healing of damaged vaginal epithelium (Takada et al., 2018). *G. vaginalis* supernatants inhibit wound healing of cervical cell monolayers compared to *L. iners* (Zevin et al., 2016). *L. crispatus*, *L. rhamnosus*, *L. gasseri*, and *L. jensenii* supernatants reduce the expression of autophagy genes ATG14, BECN1, and cyclin A, cyclin-dependent kinase 2 (CDK2), and HPV E6 and E7 to inhibit cervical cancer cells activity (Motevaseli et al., 2016; Ghanavati et al., 2020). Although most studies demonstrate the protective role of *Lactobacilli* products, lactic acid has conflicting results. Lactate and butyrate inhibit HDACs, thereby promoting histone acetylation by histone acetylases. Acetylated histones induce chromatin relaxation, resulting in an increased DNA repair rate (Wagner et al., 2015; Wagner et al., 2017), and recruitment of DNA-dependent protein kinase catalytic subunits (DNA-PKcs) to the nucleus. Cervical epithelial cells bear the lactate hydroxycarboxylic acid receptor 1 (HCA1/GPR81). Lactate stimulates surface HCA1 and induces cAMP signalling and the cAMP/EPAC/PKA-dependent shuttling of DNA-PKcs. DNA-PKcs sense foreign or damaged self-DNA and are essential components of the non-homologous end-joining pathway which protects cells from lentiviral (HPV and HIV-1) transduction, thus restricting viral oncogenic and/or cytopathic potential (Wagner et al., 2021). In addition, core fucosylation levels are significantly reduced in the serum, exfoliated cervical cells, and tumour tissues from patients with cervical cancer. *L. iners* metabolites and lactate activate the Wnt pathway by means of the lactic acid-HCA1 complex, increase the level of core fucosylation, thereby inhibiting the proliferation and migration of cervical cancer cells (Fan et al., 2021a). In contrast, lactic acid (a major metabolite in glycolytic tumour cells) can be imported to fuel mitochondrial respiration (Warburg, 1956). Lactate induces the expression of TGF-β2 in gastric and lung cancers which upregulates the expression and activation of MMP-2, thereby promoting tumour cell metastasis. It also promotes angiogenesis by upregulating HIF-1α and VEGF (Kolev et al., 2008). In addition, the immunomodulatory effect of lactate on T cells, macrophages, and CTL may lead to tumour immune escape (Colegio et al., 2014; Brand et al., 2016). Detection of high lactate levels among human cervical cancers predicted tumour metastasis, recurrence and restricted of patient survival (Walenta et al., 2000). Li et al. demonstrated that lactate induces migration and invasion of SiHa cells *via* the miR-774/ARHGAP5 axis, although the effect is diminished due to partial inhibition of E6 and E7 expression (Li et al., 2019). However, the exact roles of lactic acid in cervical cancer with respect to HPV protein expression, proliferation, and migration of cancer cells remain unclear.

### HIV infection

Most studies show that BV and low *Lactobacilli* CVM are associated with an increased risk of HIV acquisition (Atashili et al., 2008), transmission (Cohen et al., 2012), and a risk factor for intrapartum HIV trasmission(Atashili et al., 2008), and adverse pregnancy outcome(Short et al., 2020; Mwenda et al., 2021). A nested case-control study including five cohorts shows concentration-dependent associations between *Eggerthella species type 1, Gemella asaccharolytica*, *Leptotrichia/Sneathia*, *Megasphaera* spp., and *Mycoplasma hominis* with increased odds of HIV acquisition (Eastment and McClelland, 2018). Other studies also demonstrate that *Prevotella melaninogenica, V. montpellierensis, Mycoplasma, P. bivia*, *Sneathia sanguinegens* (Gosmann et al., 2017), and L.iners (Taku et al., 2022) are associated with HIV acquisition. HIV with unsuppressed viral loads are more likely to have higher abundance of *Megasphaera genomosp.1*, *A. vaginae*, and *Clostridiales* sp. compared to healthy and recurrent BV patients (Elwood et al., 2020). Relative studies are summarized in **Supplementary Table 2**.

The impact of the cervix immune response induced by the BV-associated microbiome on HIV acquisition is complicated and conflicting. On the one hand, the increase in BV-associated microbiota and inflammatory cytokines indicates a disruption of the epithelial barrier associated with HIV genital tract shedding and the recruitment of HIV target cells. Abnormally high levels of several cytokines such as IL-1β, IL-6, MIP-1α, and TNF-α are associated with HIV disease progression (Herold et al., 2013) and contribute to HIV persistence during antiretroviral treatment (ART) by promoting residual levels of viral production (Vandergeeten et al., 2012). BV-associated flora are associated with endo- (Gosmann et al., 2017) and ectocervix (Thurman et al., 2015) CD4^+^CCR5^+^T cells compared to healthy flora. Elevated inflammatory cytokine levels are associated with an increased frequency of cervical CD4^+^T cell (Arnold et al., 2016; Gosmann et al., 2017). Activated CD4^+^T cells and GD T cells are affected by vaginal flora and are associated with HIV infection. GD1 T cells are protective in the genital tract and GD2 T cells are targets for HIV entry. Cervical GD1 T cells and GD2 T cells are lower and higher in women with BV than in those with normal vaginal flora, respectively which predisposes women with HIV acquisition (Alcaide et al., 2016). On the other hand, mucosal IgG and IgM elevated with genital inflammation, IgM neutralises and reduces HIV infections and directly binds to CD4^+^T cells and chemokine receptor CCR5, hindering HIV entry (Sobia et al., 2021). Some bacteria, such as *L. gasseri*, *L. crispatus*, *V. montpellierensis*, *P. amnii*, and *S. amnii* are positively correlated with the frequency of mucosal-activated natural killer (NK) cells that coordinate the early control of HIV infection (Munusamy et al., 2021). The exact effect of CVM on HIV infection *via* local immune responses remains further explored.

CVM also plays a role in the pharmacokinetics of pre-exposure prophylaxis (PrEP) drugs. However, previous studies reported inconsistent results on this. Some clinical trials using topical 1% tenofovir (TFV) vaginal gel in the CAPRISA 004 cohort and TFV vaginal film in the FAME 04 cohort found that women with low *Lactobacilli* flora have reduced mucosal concentrations of TFV, which potentially reduces the efficacy of PrEP drugs (Klatt et al., 2017). BV cervicovaginal larvae and *G. vaginalis* reduce the uptake of PrEP drugs by Jurkat cells, and increase TFV degradation and HIV infection compared to normal cervicovaginal larvae and *L. crispatus* (Cheu et al., 2020). However, another study examined the effect of CVM on TFV vaginal ring which can continuously deliver for approximately 14 days, and showed a positively correlates between CST IVA/B and high level of TFV during the first two months of use (Thurman et al., 2022). Nevertheless, individuals with a high abundance of *Prevotella* spp. or *G. vaginalis* show reduced TFV/TFV-diphosphate concentrations in the vaginal fluid and tissues in the third month. In addition, pH drives TFV release since it affects TFV transport into human cells (Johnson et al., 2012; Taneva et al., 2018). The authors speculate that a higher pH *in vivo* increases aqueous solubility, and more TFV will dissolve from the formulated paste inside the vaginal ring, which may explain the positive correlation between TFV and CST IVA/B in the first two months.

Protonated L-lactic acid is a major anti-HIV metabolite of Lactobacilli (Kyongo et al., 2015; Ñahui et al., 2017; Tyssen et al., 2018). 0.3% w/w L-lactic acid shows a 17 times higher anti-HIV activity than that of D-lactic acid. Moreover, a physiological concentration of L-lactic acid can inactivate HIV in the presence of 50% cervix vaginal secretions and 0.75% semen. The anti-HIV activity of L-lactic acid is pH dependent, and can be abrogated under neutral pH (Aldunate et al., 2013). Healthy cervicovaginal mucus inhibits HIV activity and transmission. HIV spreads in the form of free viral particles and cellular contact. Although L-lactic acid has a stronger ability to inhibit HIV activity than D-lactic acid, the HIV-1 virion can be greatly trapped in CVM with high D-lactic acid concentration and an *L. crispatus*-dominant microbiota (Nunn et al., 2015). HIV virions have increased mobility in *L. iners* dominant mucus and BV mucus which is related to the levels of D-lactic acid and total lactic acid. The trapping of HIV-1 viruses in the cervicovaginal mucus is mainly based on adhesive interactions. The addition of lactic acid to the cervicovaginal mucus with reduced adhesive properties did not restore HIV adhesion (Nunn et al., 2015). Therefore, the reduction in mucoadhesive interactions might be due to an increased amount of mucin-degrading enzymes that Thus, the reduction in adhesive interactions may be due to elevated levels of mucin-degrading enzymes which disturb HIV-mucin interactions.

The release of extracellular vesicles (EVs) is another approach which protects HIV infection mediated by Lactobacilli (Ñahui et al., 2019). EVs constitute a lipid bilayer membrane carrying numerous bioactive molecules and are considered to be important mediators of cell–cell communication (Costantini et al., 2022; Nagakubo et al., 2019). *Lactobacillus*-derived EVs (*L. crispatus* BC3 and *L. gasseri* BC12) bind to the HIV-1 Env protein, leading to a reduction of HIV-1 entry and binding to target cells. Nevertheless, EVs released by other gram-positive species (Staphylococcus aureus, G. vaginalis, Enterococcus faecium, and Enterococcus faecalisi) also protect human cervicovaginal tissues ex vivo and isolated cells from HIV-1 infection by blocking HIV-1-cell receptor interactions which are related to the steric hindrance of gp120 or gp120 modification (Costantini et al., 2022).

### Confounders affect microbiome sequencing

Several confounders may account for the discrepancies across studies among the clinical sample sequencing researches. First, the difference in the baseline characters of subjects, such as ethnicity and geographical region, age, menstrual state, previous hormone and antibiotic treatment history can influence the composition of the reproductive tract microbiome. One example is that some studies about microbiota sequencing on CT infection show inconsistence on Aerococcus christensenii and diversity (Balle et al., 2018; Di Pietro et al., 2018; Cheong et al., 2019). Confounders such as different region, ethnicity and age may account for these bias. Second, sampling site and methodology may also have influence on microbiome sequencing as well as cytokines detection. As discussed before, there are difference in the microbiome composition of vagina wall, posterior fornix and endocervix, as well as sampling device (Kim et al., 2009; Virtanen et al., 2017; Kero et al., 2023). Cytokine and protein concentration were also different collected by endocervical swabs, lavage samples, and vaginal swabs (Dezzutti et al., 2011). Third, sequencing method, laboratory and data processing differ across studies, such as DNA extraction, sequencing target, platform, and annotation database may also impact the sequencing, metabolome and proteome result (Theodoridis et al., 2012; Schnaars et al., 2018; Mattei et al., 2019; Li and Sui, 2021; Sirichoat et al., 2021). For instance, the richness and diversity of cervical microbiota increased in HR-HPV positive women compared with healthy control by 16S rRNA sequencing, however, the difference were not obvious in metagenomic sequencing (Fang et al., 2022). Despite the discrepancies in the result of some specific bacteria in different sequencing studies due to these confounders, presence or increased relative abundances of Lactobacillus spp. are generally associated with decreased risk of female reproductive STI; BV (CST IV vaginal microbiota), and particular BVAB have been found to be associated with increased risk.

## Conclusion and outlook

Recent accumulating studies have shown complex interactions between CVM, metabolites, and host cells in the cervical ecosystem. Here, we focused on the crosstalk between CVM and cervical epithelial-, immune-, and mucus barriers.

Generally, *Lactobacillus*-dominant microbiota exhibit immunomodulatory effects on cervical epithelial and immune cells, maintain cervical epithelial integrity, and protect cervical CT, HPV, and HIV infection with respect to infection, treatment, and clearance. CVM dysbiosis induces an inflammatory response in cervical epithelial and immune cells in microbe-specific modes and directly induces epithelial barrier disruption by inducing oxidative stress, miRNA alteration, and promoting cell cycle arrest, apoptosis, and necrosis. Furthermore, an indirect response may occur through the secretion of harmful metabolites and causes immune disorders. Dysregulation of cervical cytokine and immune cell profiles induced by CVM dysbiosis is also associated with STI infection and relative disease development. *Lactobacillus* binds mucin, while BVAB stimulates mucin expression and degrades these highly glycosylated proteins which decrease the ability to trap and remove pathogens. BVABs also degrade collagen, gelatin, and casein, and alter cervical biomechanical properties which cause rapid cervical remodelling and harm human pregnancy.

Lactic acid is the most well-studied and potent normal CVM metabolite in maintaining cervicovaginal barrier homeostasis, and inhibiting CT infection and HIV activity; however, its exact role in HPV infection and cervical cancer remains unclear. The specific mechanisms involved in the *Lactobacillus*, culture supernatant and lactic acid in anti-STI infection mainly include 1) reduces polar lipids and α5β1 integrin subunit exposure in the epithelial plasma membrane, inhibit EB binding and invasion in epithelial cell; 2) inhibit HDACs so that inhibit host cell proliferation, and induce chromatin relaxation, increase DNA repair rate; 3) recruit DNA-PKcs to the nucleus, which can sense foreign or damaged self-DNA, protects cells from lentiviral (HPV and HIV-1) transduction; 4) reduce the expression of autophagy genes and HPV E6 and E7 to inhibit cervical cancer cells activity. Contradictory mechanisms mainly include 1) can be used as fuel mitochondrial respiration in glycolytic tumour cells; 2) promote MMP-2, HIF-1α, VEGF, miR-774/ARHGAP5 axis which promote metastasis, angiogenesis, migration and invasion; 3) immunomodulatory effect on T cells, macrophages, and CTL may lead to tumour immune escape. The association between BVAB, culture supernatant and SCFAs with STI infection usually related with the cytokines change and immune cells recruitment induced by the inflammatory response. The specific mechanisms in the influence on cervical STI disease are less documented.

Going forward, although some studies have observed the interactions between CVM, their metabolites, with host microenvironment using omics technology, the mechanistic understanding towards how these microbiota and their metabolites influence STI infections are lack. For instance, the bioactive metabolites which can interact with bacteria, STI pathogens and host epithelial and immune cell and are less identified, as well as the possible passways. A better understanding can provide information in the developing and optimizing of diagnostics, treatment strategies and drugs, probiotics, postbiotics and vaginal flora transplantation. The innumerable hypotheses generated from these omics studies should be verified by *in vitro* and *in vivo* studies. A major gap towards these issue is a lack of suitable *in vitro* and animal experimental models (Bjornson-Hooper et al., 2022; Mahajan et al., 2022). The microbiome, cervicovaginal epithelium and immune reaction differ between human, mice and non-human primates. Exploring suitable models simulate female reproductive tract environment is essential to better understand the intimate interaction between CVM, their metabolites and cervical host cell. Advanced tools including metagenomics, metatranscriptomics, and metabolomics will provide insights into the characteristics of functional microbiomes and metabolites, determine the potential mechanisms used to connect with host cells, and further form interaction networks in health and disease. Future studies involving molecular detection technology in human samples, cells, and animal biological studies will aid in the discovery of novel diagnostic and therapeutic targets for female reproductive diseases.

## Author contributions

MD, CH, and FX conceived the study question, and all authors were involved in the study design. MD,YD created the first draft of the manuscript. JB, HuaL, XM, BL, CW, HuiL, and AF made substantial contributions to drafting the article and revising it critically. All authors contributed to the article and approved the submitted version.
