# Supplementary material for: Interactions between microbiota and cervical epithelial, immune, and mucus barrier
Source: Front Cell Infect Microbiol. 2023 Feb 24;13:1124591. doi: 10.3389/fcimb.2023.1124591 (PMC9998931; doi:10.3389/fcimb.2023.1124591)
Supplement: Supplementary file 1 [file Table_1.docx]

| Supplementary Table 1 The clinical studies of cervicovaginal microbiota sequencing in HPV infection women | | | | | |
| --- | --- | --- | --- | --- | --- |
| Author(year) | Population | Ethnicity | Sample | Method | Findings |
| Xia et al (2022) | Healthy controls N=43,  High risk HPV+ with normal TCT or normal colposcopy or with chronic cervicitis by histopathological diagnosis of biopsy N=58 (P1 group), High risk HPV-positive with LSIL N=34 (P2 group) | Asian | Vaginal and Cervical swabs | 16S V3–V4 | *Lactobacillus* was the main genus of vaginal bacteria with or without HPV in asymptomatic women. The diversity of vaginal flora in P1 group was significantly higher than that in N group, and the proportion of *Gardnerella* was significantly increased.The structure of vaginal flora in P1 group was different from that in N group, which showed the decrease of *Lactobacillus* crispatus and the increase of *Shuttleworthia*. |
| Shi et al (2022) | HPV+/LSIL patients N=51,  HSIL patients N=22 | Asian | Cervical swabs | 16S V4–V5 | No significant differences were observed in age, disease stage, HPV subtype, vaginal microbiota community status types, and diversity (α and β) between HPV cleared and non-cleared HPV groups. Women with *enterococcus* ASV_62 depletion and *Lactobacillus* *iners* enrichment at baseline were less likely to have HPV clearance at 12 months. There was a significant negative association between high abundance of *L. iners* and HPV clearance in patients receiving non-operative treatment, but not in patients receiving surgical treatment. |
| Hu et al (2022) | HPV+ patients N=94,  HPV- patients N=182 | Asian | Cervical samples | 16S V3–V4 | The abundance of *Gardnerella* and *Atopobium* *vaginae* was significantly higher in the HPV-positive group than in the HPV-negative group, while *Burkholderiaceae* and *Mycoplasma* were more abundant in the unique-268 group (infections with one of the HPV genotype 52, 56, or 58) compared to the negative group. |
| Vikramdeo et al (2022) | 36 women with CIN：  Hispanic/Latina (HIS) women N=12,  African American (AA) women N=12, Caucasian American (CA) N=12 | Hispanic/Latina, African American and Caucasian American | Cervical biopsied samples | 16S V3–V4 | The abundance of beneficial *Lactobacillus* in AA and HIS women was significantly lower than in CA women with CIN lesions；  The abundance of *Micrococcus* was also significantly increased in AA and HIS women compared to CA women. AA ad CA women had higher levels of detection of Rhizobium compared to HIS women；  *Rubellimicrobium, Podobacter, Brevibacterium, Atopobium, Paracoccus, Brevundimonous, Comamonous,* and *Novospingobium* was detected only in the CIN lesions obtained from AA and CA women. |
| Liu et al (2022) | hrHPV+ without cervical lesion category N = 34 (HPV group), precancerous lesions with hrHPV category N= 40 (CIN group),  invasive cervical cancer category N = 41(CC group) | Asian | Cervical swabs | 16S sequencing | With the progression of cervical lesions, the diversity of cervical flora gradually increased, and the abundance of *Lactobacillus* decreased;  The expression of HPV oncogenes was observed to be positively correlated with the genera *Sneathia, Salmonella, Leptotrichia, Pseudomonas,* and *Roseovarius* in the HPV group;  In the CIN group, the enrichment of *Sneathia* and *Megasphaera* genera was weakly associated with HPV oncogene overexpression;  A strong association was also found between the excess of *Peptostreptococcus* and *Enterococcus* genera and high expression of HPV oncogenes in the CC group. |
| Yang et al (2022) | HPV+ N=12,  HPV- N=22 | Asian | Vaginal swabs | 16S rRNA sequencing | *Lactobacillus gasseri, Streptococcus agalactiae,* and *Timona prevotella* bacteria may be associated with HPV clearance. |
| Liu et al (2022) | 82 women from six ethnic groups with and without HPV Infection  Zang N = 42,  Naxi N = 13,  Yi N = 7,  Bai N = 4,  Lisu N = 6 ,  Han N = 10 | Asian | vaginal, cervical, and rectal swabs | 16S rRNA | *Lactobacillus* was dominant in most vaginal samples, declining in HPV+ samples, and varied across ethnic groups. |
| Dong et al (2022) | BV+N =316,  BV- N = 604;  HPV+N=287,  HPV- N=576 | Asian | Vaginal and Cervical swabs | 16S V3–V4 | In women with persistent HR-HPV infection and HSIL, *Prevotella* and *Gardendella* had higher abundance, while *Lactobacillus* had lower abundance;  The vaginal abundance of *Prevotella* and *Gardnerella* increased in women with persistent HPV16 infection, but only the abundance of *Prevotella* increased in women with persistent HPV18 infection;  *Prevotella* abundance in the vagina was significantly positively correlated with the expression levels of TLR4, NF-κB, C-myc and hTERT in cervical cells. |
| Fang et al (2022) | HPV+ N=20 (HR-HPV),  HPV- N=20 (Control) | Asian | Cervical swabs | 16S V3–V4 | *Firmicutes* decreased and *Actinomyces* increased during HR-HPV infection. At the generic level, *Lactobacillus* was enriched in control group, while *Gardnerella* and *Bifidobacterium* were at lower levels; *Lactobacillus crisp, Lactobacillus crispatus, L. jensenii, and L. helveticus* were enriched in control group. |
| Lopez-Filloy et al (2022) | 156 women with symptomatic cervical ectopy | Mexico | tissue biopsy and a cervicovaginal mucus sample | 16S V4 | Women with symptomatic cervical ectopy and HPV infection had increased diversity, and their vaginal microbiota was enriched in bacterial vaginosis-associated anaerobes (*Sneathia, Shuttleworthia, Prevotella, and Atopobium*) and depleted in *Lactobacillus* genus; |
| Santella et al (2022) | HPV+ N=25,  HPV- N=6 | Italy | Vaginal and Cervical swabs | 16S V3-V4-V6 | *Actinobacteria*, *Proteobacteria*, and *Bacteroides* were more representative in HPV positive patients. *Lactobacilli* is the dominant genus, in which *Lactobacilli iners, Lactobacilli jensenii,* and *Lactobacilli crispatus* account for a high proportion. *Gardnerella vaginalis, Enterococcus spp., Staphylococcus spp., Proteus spp.*, and *Atopobium* were most commonly represented in HPV-positive patients. |
| Xu et la (2022) | HPV+ N=230,  HPV- N=193 | Asian | Vaginal and Cervical swabs | 16S V3–V4 | The diversity of vaginal microbiome increased significantly after BV, HPV and BV infection with CIN group;  The enrichment of *L. iners* in HPV infection group was significant compared with other infection groups. |
| Lin et al (2022) | Normal group N = 379;  CIN1+，HPV+ N= 17,  CIN1+，HPV- N= 34 | Asian | Vaginal and Cervical swabs | 16S V4 | Patients with BV had a higher prevalence of HR-HPV;  Women with HR-HPV infection and cervical intraepithelial neoplasia (CIN) had higher positive rates of sialase (SNA);  α diversity was significantly higher in HPV+ women than in HPV- women;  The proportions of Gardnerella and Prevotella increased significantly in HPV (+) patients. |
| Liu et al (2022) | HSIL N = 18,  SCC N = 30,  postmenopausal normal women N = 30 | Asian | Vaginal and Cervical swabs | 16S rRNA | With the progression of the disease, the expression level of *Lactobacillus iners* and *Lactobacillus* total vagina decreased gradually; |
| Mei et la (2022) | HR-HPV persistent infection (P group) N = 28,  HR-HPV (C group) N = 30,  no history of any HR-HPV infection (NC group) N = 42 | Asian | Vaginal and Cervical swabs | 16S V3–V4 | Compared with NC group, P and C groups showed an increase in *Firmicutes* and *Actinobacteriota,* but a decrease in *Proteobacteria*;  *Proteobacteria* phylum was significantly different in the NC group, but no indicative taxa were found in the P and C groups;  *Bifidobacterium* and *Lactobacillus* in C group had higher mean relative abundance than in the NC group; |
| Nieves-Ramirez et al (2021) | 121 participants with SIL, most of which were HPV positive,  HPV- N=107 | Mexico | Vaginal and Cervical swabs | 16S V3 | HPV infection was independently associated with an increase in the relative abundance of *Brachybacterium conglomeratum* and *Brevibacterium aureum* and a decrease in two *Lactobacillus iners* OTUs;  Positive independent association between HPV-16 and *Brachybacterium* *conglomeratum*. |
| Wang et al (2022) | locally advanced cervical cancer patients before neoadjuvant chemotherapy (LACC) n = 26,  healthy controls N = 40 | Asian | Vaginal swabs | 16S V3–V4 | Compared with healthy controls, α diversity was significantly increased in CC patients and more non-conventional bacteria colonized;  There were also significant differences in β diversity between cervical cancer patients and control group;  In patients with CC, α diversity in vaginal samples was significantly higher in non-responders than in responders ; |
| Zhang et al (2021) | HPV16/18(+) and cervical carcinoma N = 10,  HPV16/18(+) but no cervical carcinoma N = 38,  other hrHPV(+) N = 32,  healthy controls with HPV(-) N = 20 | Asian | Vaginal and Cervical swabs | 16S V3–V4 | The cervical microbiome consists of *Firmicutes* below the level of the vaginal hilum and a higher percentage of *Proteobacteria*;  Sphingotoxin belongs to α-Proteobacteria, which is almost below the vaginal detection limit, but accounts for 5% to 10% of hrHPV (-) cervical bacteria and were negatively associated with hrHPV infection;  *Pseudomonas* belongs to the *γ-Proteobacteria*, which is almost invisible in the normal vagina and accounts for a small percentage of the normal cervix, but is found in HPV16/18 (+) and cancerous cervix were significantly higher; |
| Wei et al (2020) | HPV+ N=30,  HPV- N=30 | Asian | Vaginal and Cervical swabs | 16S V3–V4 | Microbial perturbation occurs in the early stages of hr-HPV infection;  *Lactobacillus* and *Sporolactobacillus* decreased, while bacteria associated with BV such as *Gardnerella, Prevotella, Dialister, Slackia, Actinomyces, Porphyromonas, Peptoniphilus, Anaerococcus, Peptostreptococcus, Streptococcus, Ureaplasma, Megasphaera,* and *Mycoplasma increased.* |
| Xie et al (2020) | healthy women (C group) N = 30, cervical intraepithelial neoplasia patients (CIN group) N = 30  cervical cancer patients (CER group) N = 30 | Asian | Vaginal and Cervical swabs | 16S V4 | Cervical cancer was strongly associated with a decrease in the probiotic *Lactobacillus* and an increase in the pathogen *Prevotella spp., Sneathia spp.* and *Pseudomonas spp.* |
| Zhou et al (2020) | 176 women, either with VAIN, or without VAIN but with HPV infection | Asian | Vaginal swabs | 16S V4 | VAIN's vaginal microbiome is characterized by increased abundance of *Atopobium, Gardnerella, Allobaculum* and *Clostridium,* and decreased abundance of *Finegoldia, Actinobaculum* and *Blautia*. Higher levels of *Enterococcus* and certain types of *Clostridium spp.* might be associated with an elevated risk of VAIN2/3 |
| Liu et al (2020) | HPV+ N=91,  HPV- N=31 | Asian | Vaginal swabs | 16S rRNA | Reduced levels of probiotics, including *Shuttleworthia, Prevotella, Lactobacillus,* and *Sneathia*, and increased levels of pathogenic bacteria, including *Dispar, Streptococcus*, and *Faecalibacterium prausnitzii,* may be a direct result of early HPV infection;  Other disease-causing bacteria, such as *Bifidobacteriaceae,* may be key factors in cancer progression; |
| Qingqing, B et al (2021) | HPV persistent infection N = 6,  HPV transient infection N = 4,  Health control N = 5 | Asian | cervical specimens | 16S rRNA | In healthy and transient infection individuals, the structure of the cervicovaginal microbiome is relatively simple, and *Firmicutes* dominate;  HPV persistent infection showed a complex trend, with high abundance of *Proteobacteria, Actinobacteria, Bacteroidetes* and *Fusobacteria;*  *Prevotella, Sphingomonas* and *Anaerococcus* were associated with persistent HPV infection;  *Lactobacillus iners* is associated with transient HPV infection; |
| Cheng et al (2020) | visited a youth clinic N = 169,  cervical screening N = 88 | Sweden | Vaginal and Cervical swabs | 16S V3–V4 | Microbial α diversity was significantly higher in the HPV-infected group (especially carcinogenic HPV types and multiple HPV types) compared to the HPV-negative group;  HPV infects the vaginal microbiome of women characterized by a large number of bacterial vaginosis associated bacteria (BVAB), *Sneathia, Prevotella,* and *Megasphaera*;  Twice as many women were infected with non-*Lactobacillus*-dominant vaginal microbiota that were infected with carcinogenic HPV types, compared with *L. crispatus*. |
| So et al (2020) | HPV+,without CIN N=10  HPV+, CIN N1=10  HPV+, CIN N2/3=10  HPV+, invasive squamous cell carcinoma N=10  HPV- N=10 | Korea | cervicovaginal swab | 16S V3-V4 | The CIN/cancer group had significantly fewer *Lactobacillus crispatus* than the normal group;  Increased microbial diversity in patients with CIN or cervical cancer wiht HPV infection;  *Atopobium vaginae, Dialister invisus, Finegoldia magna, Gardnerella vaginalis Prevotella buccalis,* and *Prevotella* *timonensi*s were significantly associated with CIN 2/3 or cervical cancer risk. |
| McKee et al (2020) | women with abnormal cervical cytology N = 109 (ASC N = 55, LSIL N = 45, HSIL N = 6, AGC N = 3),  HPV+ N = 110,  NILM/HPV− N = 89 | Mix | Vaginal and Cervical swabs | 16S V3–V4 | Women with abnormalities in cervical cytology or HPV+ were more likely to have a diverse vaginal microbiome characterized by a relatively high abundance of *Gardnerella vaginalis*, while women without abnormalities in cytology were more likely to be *Lactobacillus spp*. Dominate;  Women without cytological abnormalities predominate in the community with a higher prevalence of *L. iners* than women with abnormal cervical cytology and HR HPV+ abnormalities;  The relative abundance of *L. gasseri* varied more in these women than in women with cervical cytological abnormalities or at high risk for HPV+. |
| Chen et al (2020) | HPV+ without CINs N = 78(HPV group) ,  LSIL group N = 51,  HSIL group N = 23,  Cancer group N = 9 ,  HPV - N = 68 (Normal group) | Asian | Vaginal and Cervical swabs | 16S V3–V4 | HPV infection increases the richness and diversity of vaginal bacteria, regardless of CIN status;  HPV infection negatively affects the abundance of *Lactobacillus*, *Gardnerella* and *Atopobium*;  HPV infection increases the relative abundance of *Prevotella, Bacillus, Anaerococcus, Sneathia, Megasphaera, Streptococcus* and *Anaerococcus*;  HPV infection without CIN or cancerous lesions is closely associated with *Megasphaera*;  The most abundant bacteria in the LSIL group was *Prevotella amnii*;  The familial level of *Prevotella timonensis, Shuttleworthia* and *Streptococcaceae* are the three groups associated with HSIL;  Cancer group: *Bacillus, Sneathia, Acidovorax, Oceanobacillus profundus, Fusobacterium, Veillonellaceae* increased*.* |
| Yang et al (2020) | HPV16 positive women N = 27  age-matched HPV negative controls N = 25 | Asian | Vaginal and Cervical swabs | 16S rRNA | The relative abundance of dominant Firmicutes was lower in HPV16 positive group, while the relative abundance of *Actinobacteria, Fusobacteria* and viruses phyla were significantly higher;  Seventy-seven genera, including *Gardnerella, Peptostreptococcus,* and *Prevotella*, were lower in HPV16-positive women, and 20 genera, including *Lactobacillus* and *Aerococcus*, were lower. |
| Wu et al (2020) | NILM N=31,  LSIL N=22 ,  HSIL N=16 | Asian | Vaginal swabs | 16S V3–V4 | *Prevotella* and *streptococcus* increased in group HSIL;  Squamous intraepithelial neoplasia transforms the bacterial community structure of the vagina from CSTs IV to II;  Microbiota diversity was more pronounced in CST types II and IV, especially in type II;  *Peptostreptococcaceae* family, *Pseudomonadales* order, and other bacteria types were significantly higher in women without intraepithelial lesions or malignancies than in women with squamous intraepithelial neoplasia.  Delftia was enriched in the LSIL and HSIL groups compared with those without intraepithelial lesions or malignancies. |
| Mitra et al (2020) | CIN 2 N =95 | Mix | Cervical swabs | 16S V1–V2 | Women with a *Lactobacillus*-dominated microbiome at baseline were more likely to have degenerative disease at 12 months;  Depletion of *Lactobacillus spp*. and the presence of specific anaerobic groups (including *Megasphaera, Prevotella timonensis* and *Gardnerella vaginalis*) are associated with CIN2 persistence and slower resolution. |
| Usyk et al (2020) | two visits of women with an incident HR-HPV infection N = 273 women | Costa Rica | Cervical swabs | 16S V4 | *Lactobacillus*, V1 fungal diversity, and V1 functional Cell Motility pathwa showed significant protective effects;  Bacterial diversity was shown to predict progression to CIN2+. |
| Borgogna et al (2020) | HPV+ N=26,  HPV- N=13 | United States | Vaginal and Cervical swabs | 16S V1-3 | The vaginal metabolome of women with HPV infected differs from women with HPV in several metabolites, includingbiogenic amines, glutathione, and lipid-related metabolites. |
| Zhou et al (2019) | Low-risk HPV positive group N = 42, (LR group ),  negative control N = 20 (NC group) | Asian | Vaginal swabs | 16S V3–V4 | The relative abundance of *Firmicutes* potentialis was low with LR-HPV infection, while the relative abundance of *actinomyces, Proteus* and *Clostridium* was obviously high;  At the generic level, *Gardnerella, Bifidobacterium, Sneella, hydrophilus, Burkholderia*, and *Atopodella* were higher in the LR group. |
| Chen et al (2019) | hrHPV-infected pregnant women N = 38 (PHR group), pregnant women without HPV infection N = 48 (PN group), nonpregnant women with hrHPV infection N = 19 (NPHR group) and nonpregnant women without HPV infection N = 30 (NPN group) | Asian | Vaginal and Cervical swabs | 16S V3–V4 | Both pregnancy and hrHPV infection were accompanied by increased rates of CST I (dominated by *Lactobacillus* *crispatus*) but not CST III;  *Bifidobacterium, Bacillus, Megasphaera, Sneathia, Prevotella, Gardnerella, Fastidiosipila* and *Dialister* were found to be biomarkers for hrHPV infection in women;  *Bifidobacterium, Megasphaera, Bacillus, Acidovorax, Oceanobacillus* and *Lactococcus* have been associated with hrHPV infection in pregnant women. |
| Ilhan et al (2019) | healthy HPV-negative N = 18 (Ctrl HPV− group),  HPV-positive participants N = 11 (Ctrl HPV+ group),  LSIL , N = 12,  HSIL N = 27,  invasive cervical carcinoma N = 10 (ICC group) | Mix | Cervicovaginal lavages and vaginal swabs | 16S rRNA | Adenosine and cytosine were positively correlated with *Lactobacillus* abundance and negatively correlated with genital inflammation. |
| Onywera et al (2019) | HPV+ N = 37,  HPV- N = 30 | Black South African | Cervical swabs- | 16S V3–V4 | Compared to women with low risk (LR) -HPV or no HPV infection, women with HR-HPV had significantly higher relative abundances of *Aerococcaceae*, *Pseudomonadaceae* and *Bifidobacteriaceae* ;  The relative abundance of *Gardnerella, Sneathia,* and *Atopobium* was also higher in HR-HpV-infected women compared to women with low risk (LR) -HPV or no HPV infection. |
| Chao et al (2019) | HPV+ N = 65,  HPV- N = 86 | Asian | Vaginal and Cervical swabs | 16S V4 | Anaerobic bacteria such as *Bacteroides plebeius, Acinetobacter lwoffii,* and *Prevotella buccae* were found significantly more frequently in HPV-positive women;  There were no significant differences in *L. iners, L. jensenii,* and *L. gasseri*. |
| Kwasniewski et al (2018) | Control group and HPV(‑) N=70, LSIL and HPV(‑) N=95,  HSIL and HPV(+) N=85 | Poland | Vaginal and Cervical swabs | 16S V4 | The volunteers' CST cervical swabs were characterized by *Lactobacillus crispatus, Lactobacillus iners* and *Lactobacillus taiwanensis*, but not by *Gardnerella vaginalis* and *Lactobacillus acidophilus*;  In the CST of LSIL patients, the main bacterial types were *Lactobacillus acidophilus* and *Lactobacillus iners,* but *Lactobacillus crispatus* was not detected;  CST of women with HSIL showed abundance of *Gardnerella vaginalis* and *Lactobacillus acidophilus*, but lack of *Lactobacillus taiwanensis, Lactobacillus iners* and *Lactobacillus crispatus*. |
| Ritu et al (2019) | HPV+ N = 90,  HPV- N = 43 | Asian | Cervical swabs | 16S V4 | HPV positive women had lower cervical microbial richness than HPV negative women  *Ureaplasma parvum* and related taxa were associated with baseline HPV positivity, while *Brochothrix, Diplorickettsia, Ezakiella, Faecalibacterium,* and *Fusobacterium* genera were associated with baseline HPV negative;  For HPV-positive women, baseline abundance of *Actinomyces* was negatively associated with new HPV infection, *Alloprevotella tannerae, Prevotella nigrescens*, and *Prevotella oulorum*;  There was a positive correlation between *Dialister invisus* and the number of new HPV infections in the follow-up years;  *Lactobacillus delbrueckii* was found to be negatively associated with persistent HPV infection, and nine taxa belonging to *Prevotella, Dialister*, and *Lachnospiraceae* were found to be positively correlated with persistence and/or negatively correlated with clearance of HPV types. |
| Godoy-Vitorino et al (2018) | HPV+ N = 52,  HPV- N = 10 | Hispanics | vaginal, cervical, and anal swabs | 16S V4 and ITS-2 fungal | In patients with CIN3 lesions, *Atopobium vaginae* and *Gardnerella vaginalis* were enriched;  Fungal diversity was significantly higher in high-risk HPV cervical samples and patients with ASCUS;  Fungal biomarker characteristics of the vagina and cervix include *Sporidiobolaceae* and *Sacharomyces* of ASCUS, and *Malassezia* for high-risk HPV infections. |
| Zhang et al (2018) | CIN 1- (normal cytology and CIN 1) N = 126  CIN 2+ (CIN 2 and CIN 3) N = 40 | Asian | cervical biopsy specimens | 16S V3–V4 | The direct and indirect associations between CIN state and *Ps. stutzeri* are in opposite directions;  The direct and indirect associations between CIN status and *A. vaginae* were the same. *B. fragilis, L. delbrueckii*, and *S. agalactiae* are only indirectly associated with CIN state. |
| Di Pietro et al (2018) | Healthy N=7,  CT N=7,  HPV/CT N=3,  HPV N=8 | Italian | Cervical swabs | 16S V3–V4 | Cervical microbiota of women co-infected with HPV/ CT showed higher microbial diversity than those of healthy controls;  *Aerococcus christensenii* is associated with CT infection. |
| Di Paola et al（2017） | HPV+ N = 55,  HPV- N = 17 | Caucasian | Vaginal and Cervical swabs | 16S V3–V5 | CST IV-BV is a risk factor for HPV persistence |
| Shannon et al（2017） | HPV + N = 23,  HPV- N = 36 | African/Caribbean | vaginal swabs and endocervical cytobrushes | 16S V3–V4 | HPV patients more frequently had a highly diverse cervicovaginal microbiome (CST IV) and were less likely to have a microbiome dominated by *L. gasseri* |
| Audirac-Chalifour et al（2016） | non-cervical lesions (NCL: N = 10 HPV-; N = 10 HPV+),  SILs (N = 4 HPV-positive)  CC (N = 8 HPV-positive). | Mexican mestizo | Cervical swabs | 16S V3–V4 | There was a significant difference in microbiome diversity between NCL-HPV-negative women and women with SIL and CC;  The dominant bacteria in cytologically normal women were *L. crispatus* and *L. iners*, while for SIL it was *Sneathia spp.*, and for CC it was *Fusobacterium spp*.; |
| Piyathilake et al（2016） | CIN 2 N= 208,  CIN 3 N= 132 ,  CIN 1 N = 90 | The majority were non-Hispanic black | Cervical swabs | 16S V4 | Cervical mucosa community type dominated by *L. iners* and unclassified *Lactobacillus spp* was associated with CIN 2+;  *Lactobacillaceae, Lactobacillus, L. reuteri*, and several sub-genus level *Lactobacillus* operational taxonomic units were also associated with CIN 2+. |
| Mitra et al（2015） | LSIL N = 52,  HSIL N= 92,  invasive cervical cancer (ICC) N = 5, healthy controls N = 20 | Caucasian/Asian /Black | Vaginal and Cervical swabs | 16S rRNA | An increase in disease severity was associated with a decrease in the relative abundance of *Lactobacillus spp*;  In contrast to LSIL, the HSIL vaginal microbiome is characterized by *Sneathia sanguinegens, Anaerococcus tetradius* and *Peptostreptococcus anaerobius* while *Lactobacillus jensenii* was low. |
| Dareng et al（2016） | HPV+ N = 66,  HPV- N = 212 | Nigeria | Vaginal and Cervical swabs | 16S V4 | hrHPV infection is associated with a reduced abundance of *Lactobacillus sp*. and an increased abundance of *anaerobes, particularly Prevotella* and *Leptotrichia* |
| Oh et al（2015） | women with CIN N = 70  control women N = 50 | Korea | cervix brush | 16S rRNA | The patterns dominated by *Atopobium vaginae, Gardnerella vaginalis* and *Lactobacillus iners* and a few *Lactobacillus crispatus* had higher risk of CIN  The abundance of *A. vaginae* had higher risk of CIN  *A. vaginae, G. vaginalis* and *L. iners* predominate, while the absence of *L. crispatus* in the cervical microbiome is associated with CIN risk |

LSIL, low-grade squamous intraepithelial lesions, HSIL high-grade squamous intraepithelial lesions**,** SCC squamous cell carcinoma, VaIN Vaginal intraepithelial neoplasia
